# Suppression of ASNS expression by VHL-mediated ubiquitination hinders the progression of renal cell carcinoma through enhancing JUP expression and inhibiting PI3K-AKT and MAPK pathways

**DOI:** 10.7150/ijbs.129332

**Published:** 2026-02-26

**Authors:** Wuping Yang, Tao Liu, Wenwei Ying, Minghao Lu, Dan Xia, Tianyu Tang, Ding Peng

**Affiliations:** 1 Department of Urology, The First Affiliated Hospital, Zhejiang University School of Medicine, Hangzhou 310003, China.; 2 Department of Urology, Peking University First Hospital, Beijing 100034, China.; 3 Department of Urology, Zhejiang Provincial People's Hospital (Affiliated People's Hospital), Hangzhou Medical College, Hangzhou 310000, China.

**Keywords:** VHL, renal cell carcinoma, L-Asparagine, L-Asparagine synthase, junction plakoglobin

## Abstract

Renal cell carcinoma (RCC) is a metabolic disorder and *VHL* gene inactivation is recognized as a crucial event in RCC progression. Investigating the specific metabolite that differ in *VHL-*mutant RCC and understanding how VHL regulates the metabolite may offer new insights into the underlying mechanisms of RCC. First, we employed untargeted metabolomics and ELISA to identify and confirm the differential metabolite in the plasma and tumor tissues of *VHL-*mutant RCC patients. Then, we demonstrated the importance of the differential metabolite in RCC progression through cell phenotype and animal experiments. Finally, we utilized western blotting, immunoprecipitation, ubiquitination modification proteomics, TMT proteomics, and RNA sequencing to elucidate the regulatory mechanisms of VHL on the metabolite. By analyzing the metabolomics data from plasma and tumor tissues alongside subsequent expression validation, we identified L-Asparagine (L-Asn) as the differential metabolite in *VHL-*mutant RCC, with its levels significantly decreased in these tumors. L-Asn was found to promote the growth and metastasis of RCC cell lines and mouse orthotopic renal tumors. Mechanistically, VHL interacted with L-Asparagine synthase (ASNS) and facilitated its ubiquitination, leading to decreased ASNS expression, and ASNS overexpression activated PI3K-AKT and MAPK signaling pathways by binding to Junction plakoglobin (JUP) and inhibiting its expression. Conversely, use of an ASNS inhibitor significantly restrained the growth and metastasis of RCC cells *in vitro* and *in vivo*. In summary, our findings highlighted the critical role of L-Asn in RCC and identified ASNS as a novel substrate for VHL-mediated ubiquitination, presenting a potential new target for RCC treatment.

## Introduction

Renal cancer ranks among the ten most common cancers worldwide, with renal cell carcinoma (RCC) being the most frequent type, representing over 90% of renal cancer cases. Clear cell RCC (ccRCC) accounts for 75-80% of all RCC instances 1, 2. RCC generally shows resistance to radiotherapy and chemotherapy, making surgery the only viable option for patients with early-stage, localized tumors. Consequently, those with advanced tumors often depend on drug therapies 3. In the last ten years, progress in targeted therapies and immunotherapies, including tyrosine kinase receptors inhibitors (TKIs) and immune checkpoint blockades (PD-1/PD-L1 inhibitors), has significantly improved outcomes for patients with advanced RCC 4, 5. However, these treatment options still face numerous challenges. Firstly, advanced RCC patients frequently develop resistance to targeted therapies 6. Secondly, the absence of reliable predictive biomarkers complicates the ability to forecast how RCC patients will respond to immune checkpoint blockades 7. Thus, it is crucial to continue searching for effective biomarkers and targets to aid in the development of new drugs and enhance patient survival rates.

The loss or mutation of the Von Hippel Lindau (*VHL*) gene is often seen as a critical early event in the development and progression of RCC, with up to 92% of ccRCC cases showing inactivated *VHL* genes 8. The VHL protein, an E3 ubiquitin ligase, is primarily recognized for its role in ubiquitinating prolyl-hydroxylated transcription factors, specifically hypoxia-inducible factors (HIFs), which leads to their degradation through the proteolytic pathway under normal oxygen conditions 9. HIF-1α and HIF-2α regulate the expression of various genes involved in angiogenesis, metabolism, chromatin remodeling, and other processes related to RCC development and progression 10. Given this biological aspect of RCC, existing targeted therapies in clinical use focus on HIF-1α, HIF-2α, or their downstream targets 11. Since the VHL protein functions as a tumor suppressor in RCC by ubiquitinating HIFs, investigating signaling pathways beyond the traditional “VHL-HIFs” pathway may offer new insights into RCC pathogenesis and help identify additional downstream targets.

The development or advancement of tumors can alter the normal metabolic processes of body, leading to changes in various metabolic indicators 12. As a result, metabolic disorders are significant indicators of cancer. Metabolomics, a rapidly advancing area in biomedical research, allows for the analysis of the overall composition and variations of metabolites within the body, providing insights into the physiological and pathological conditions of the organism. In cancer research, extensive studies in metabolomics have transformed the previous understanding of cancer metabolism, emphasizing the critical role of these metabolic alterations in tumor formation and progression, rather than viewing them merely as by-products of tumors 13, 14. RCC is also recognized as a metabolic disease, with its progression closely linked to metabolic reprogramming 15. A recent study conducted metabolomics analysis on the plasma samples of 920 RCC patients and 760 healthy control individuals. It identified seven key plasma metabolites (2-hydroxyphenylacetic acid, azelaic acid, N,N-dimethylglycine, N-acetyl-L-aspartic acid, N-epsilon-acetyl-L-lysine, proline, and (Z,Z)-4-oxo-2,5-hetpadienedioic acid), and a promising artificial intelligence-based plasma metabolism model RCAID was developed used these seven metabolites, which has potential clinical implications for the diagnosis of RCC 16. However, in addition to identifying the unique metabolites in RCC, further research to explore the biological functions and regulatory mechanisms of these differential metabolites will be able to provide a new perspective on the onset and development of RCC.

This study aims to perform metabolomics analysis on the plasma and tumor tissue samples of *VHL-*mutant RCC patients to pinpoint the specific differential metabolite in *VHL-*mutant RCC patients. Following this, the biological functions of the metabolite will be examined at both cellular and animal levels. Finally, a series of molecular biology experiments will be conducted to investigate the relationships between the specific metabolite and VHL protein, as well as the downstream molecular regulatory mechanism involved.

## Materials and Methods

### Untargeted metabolomics analysis

Blood samples were collected from healthy control individuals and patients with renal cell carcinoma (RCC) harboring *VHL* mutations, and *VHL-*mutant RCC tissues and their adjacent normal (AN) tissues were also obtained. The *VHL* gene mutation status in RCC samples was determined using the Sanger sequencing method. This study included 63 *VHL-*mutant RCC patients, including 2 frameshift mutations, 37 missense mutations, 5 nonsense mutations, 6 micro-deletions, and 13 large deletions.

Untargeted metabolomics analysis of human plasma samples (63 patients with *VHL-*mutant RCC and 31 healthy control individuals) and tissue samples (19 pairs of fresh *VHL-*mutant RCC and AN tissues) was conducted by Shanghai Baiqu Biomedical Technology Co., Ltd. using the UHPLC-QE-MS method, and the detection process has been described in detail in our previously published article 17. The orthogonal projections to latent structures-discriminant analysis (OPLS-DA) method was used to analyze the original data. The SIMCA software (V16.0.2, Umea, Sweden) was utilized to perform logarithmic (LOG) conversion and UV formatting on the data. Firstly, OPLS-DA modeling analysis was performed on the first principal component, and the quality of the model was tested using 7-fold cross validation; Then evaluated the effectiveness of the model using the R^2^Y (interpretability of the model for categorical variable Y) and Q^2^ (predictability of the model) obtained through cross validation; Finally, a permutation test was conducted to randomly change the order of categorical variable Y multiple times to obtain different random Q^2^ values, which further tested the effectiveness of the model. In the end, the multivariate statistical analysis method was utilized to analyze the data. The analysis criteria included a P-value from the Student's t-test of less than 0.05 and a Variable Importance in the Projection (VIP) score greater than 1 for the first principal component of the OPLS-DA model. In addition, the metabolomics data have been deposited in the OMIX, China National Center for Bioinformation/Beijing Institute of Genomics, Chinese Academy of Sciences (https://ngdc.cncb.ac.cn/omix: accession no.OMIX005906).

### ELISA

The levels of L-Asparagine (L-Asn), L-Asparaginase (L-ASNase), L-Asparagine synthase (ASNS), LysoPA, Indoxyl sulfate, L-Malic acid, and Ethyl glucuronide in the plasma and tumor tissue samples of *VHL-*mutant RCC patients and RCC cell lines were quantified using the ELISA method, separately following the instructions provided with the ELISA kits obtained from Shanghai Saipeisen Biotechnology Co., Ltd, Shanghai Qiyi Biotechnology Co., Ltd, Shanghai Tongwei Industrial Co., Ltd, Wuhan Yipu Biotechnology Co., Ltd., Shanghai Jichun Industrial Co., Ltd., Shanghai Qichen Biotechnology Co., Ltd., and Tianjin Tailian Biotechnology Co., Ltd.

### Cell culture and transfection

Six RCC cell lines A498, OSRC2, Caki-1 (*VHL* wild-type, metastatic RCC cell line), 769-P, 786-O (*VHL* mutant), RCC4 (*VHL* mutant) and two control cell lines HK2 (Epithelial cells of human renal cortex proximal tubules), HEK-293 (Human embryonic kidney cells) were purchased from the ATCC (American Type Culture Collection) cell bank. During the cell resuscitation process, the cells were cultured using serum-containing DMEM medium. During the experimental stage, the medium was then changed to a serum-free one. All cell lines have undergone STR authentication. Various plasmids were constructed, including VHL overexpression, VHL overexpression (with 3xFLAG tag), VHL shRNA, VHL sgRNA, ASNS overexpression, ASNS overexpression (with 3xFLAG tag), Ub overexpression (with HA tag), five ASNS protein truncation variants (amino acids 1-203, 204-385, 386-561, 1-385, 204-561, with 3xFLAG tag), ASNS mutant (AAA-AGA, 510 lysine-arginine), ASNS mutant (AAA-AGA, 510 lysine-arginine, with 3xFLAG tag), ASNS shRNA, ASNS sgRNA, and JUP overexpression plasmids. All lentiviruses were packaged using a three-plasmid system, and Lipofectamine 3000 was utilized for transfection. The specific target sequences for VHL shRNA and sgRNA were TATCACACTGCCAGTGTATAC, while the ASNS shRNA and sgRNA target sequence was GCTGTATGTTCAGAAGCTAAA. The primer sequences for the ASNS mutant plasmid were: forward primer: CCTAGAACCAAAGAAGGATATTACTACCGTCA; reverse primer: CCTTCTTTGGTTCTAGGAGTATTGAAGGGAAATTTCTGG.

### Colony formation assay

HK2, Caki-1, and 786-O cell lines were seeded into 6-well plates and then cultured in DMEM medium without serum. Subsequently, different concentrations of L-Asn or ASNS inhibitor (Bisabosqual A, 10.7μM) were added. After approximately two weeks of culture, the cells were fixed and stained with 0.5% crystal violet. Following thorough washing and drying, images were scanned, and cell colonies were counted under an inverted microscope.

### Cell migration and invasion experiments

A mixture of 200μL of serum-free cell culture medium and a suitable quantity of HK2, Caki-1 and 786-O cell lines was placed in the upper chamber, while 800μL of DMEM medium containing L-Asn or L-Asn + ASNase or ASNS inhibitor (Bisabosqual A, 10.7μM) but without serum was added to the lower chamber. After incubation of 48 hours, cells that migrated to the underside of the luminal membrane were rinsed with distilled water, fixed, and stained with 0.5% crystal violet. Following thorough washing and drying, images were captured using an inverted microscope for further analysis. The procedure for the cell invasion assay was identical, except that the upper chamber was precoated with Matrigel.

### Bioinformatics data mining

The mRNA expression data for ASNS and JUP were sourced from the TCGA-KIRC (The Cancer Genome Atlas-Kidney Renal Clear Cell Carcinoma), TCGA-KIRP (Kidney Renal Papillary Cell Carcinoma‌), TCGA-KICH (Kidney Chromophobe Carcinoma‌) datasets (https://www.cancer.gov/ccg/research/genome-sequencing/tcga), along with the clinicopathological and survival information of the patients.

### Immunohistochemical staining

The protein expression levels of ASNS and JUP in paraffin-embedded sections of RCCs and their AN tissues from our center were assessed using immunohistochemical staining. Additionally, we evaluated the protein expression of Cleaved-Caspase 3 in mouse renal tumors and the expression of E-cadherin and N-cadherin in mouse lung metastases. The specific primary antibodies utilized included: Anti-ASNS (92479, CST, 1:500), anti-JUP (11146-1-AP, Proteintech, 1:100), anti-Cleaved-Caspase 3 (9661, CST, 1:400), anti-E-cadherin (ab40772, Abcam, 1:500), and anti-N-cadherin (ab19348, Abcam, 1:100).

### Western blotting

Total protein was first extracted from various cell lines using RIPA lysis buffer, followed by protein quantification. Then, after electrophoresis, membrane transfer, and blocking, the membranes were incubated with primary antibodies overnight at 4°C. The primary antibodies used included: anti-ASNS (CST, 92479, 1:1000), anti-VHL (CST, 68547, 1:1000), anti-Ubiquitin (Abclonal, A19686, 1:2000), anti-HA-Tag (Abclonal, AE105, 1:10000), anti-FLAG-Tag (Abclonal, AE092, 1:10000), anti-JUP (Proteintech, 11146-1-AP, 1:1000), anti-AKT (Proteintech, 10176-2-AP, 1:5000), anti-p-AKT (Proteintech, 66444-1-Ig, 1:4000), anti-PI3K (Proteintech, 60225-1-Ig, 1:10000), anti-p-PI3K (Abcam, ab278545, 1:1000), anti-ERK1/2 (HUABIO, SA43-03, 1:10000), anti-pERK1/2 (HUABIO, ET1610-13, 1:5000), anti-Cleaved-Caspase 3 (CST, 9661, 1:1000), anti-Bcl-2 (Proteintech, 12789-1-AP, 1:2000), anti-BAX (Proteintech, 50599-2-Ig, 1:20000), anti-E-cadherin (Abcam, ab40772, 1:10000), anti-N-cadherin (Abcam, ab76011, 1:5000), anti-SNAIL (HUABIO, ER1706-22, 1:5000), and β-actin (Proteintech, 66009-1-Ig, 1:20000). The next day, the membranes were incubated with secondary antibodies for 2 hours at room temperature. After washing the PVDF membrane, enhanced chemiluminescence was performed.

### Detection of protein stability

CHX (Cycloheximide, 100ug/mL) was used to treat HEK-293, 786-O, RCC4, and Caki-1 cell lines in order to observe the change in the stability of ASNS protein. Then, under the continuous CHX treatment condition, 786-O and RCC4 cell lines, as well as VHL-overexpressing 786-O and RCC4 cell lines, were treated with MG132 (20uM). Subsequently, the change in ASNS protein expression was compared with the MG132 untreated 786-O and RCC4 cell lines and VHL-overexpressing 786-O and RCC4 cell lines. Next, under continuous CHX treatment, MG132 treatment was applied to HEK-293 and Caki-1 cell lines, as well as VHL-knockout HEK-293 and Caki-1 cell lines. The change in ASNS protein expression was also compared between MG132 treated and MG132 untreated HEK-293 and Caki-1 cell lines, as well as the VHL-knockout HEK-293 and Caki-1 cell lines.

### Quantitative real-time PCR (qRT-PCR)

Total RNA from the cells was extracted using the TRIzol method, and cDNA was synthesized using the RevertAid First-strand cDNA Synthesis Kit (TransGen Biotech, China). Subsequently, qRT-PCR was conducted following the manufacturer's instructions, with normalization to GAPDH. The ASNS primer sequence is: Forward primer: GGAAGACAGCCCCGATTTACT; Reverse primer: AGCACGAACTGTTGTAATGTCA.

### Immunoprecipitation (IP)

Immunoprecipitation (IP) assays were performed to validate the interaction between VHL and ASNS proteins, as well as the interaction between ASNS and JUP proteins. First, protein lysates were extracted from cells using an immunoprecipitation lysis buffer, and a portion of the lysate was reserved as the Input control. Subsequently, Anti-FLAG M2 magnetic beads (M8823, Millipore, 1:20) were added to the remaining protein supernatant and incubated overnight at 4°C. Alternatively, the target protein antibody was added to the remaining protein supernatant, incubated at room temperature for 4 hours, followed by the addition of protein A/G magnetic beads (Sigma, 1:20) and overnight incubation at 4°C. The following day, the magnetic beads were washed three times with IP lysis buffer, and then 100μl of 2× protein loading buffer was added. The mixture was boiled at 100°C for 5 minutes, after which the protein loading buffer was collected and the magnetic beads were discarded. The binding status of the target protein was then analyzed using western blotting, or the potential proteins in the differential protein bands identified through silver staining were examined using protein mass spectrometry.

### Ubiquitination modification proteomics detection

Ubiquitination modification proteomics was employed to identify differential ubiquitination modification sites in VHL-overexpressing 786-O cells compared to the control 786-O cells. This testing and analysis were performed by Hangzhou Jingjie Biotechnology Co., Ltd. The differential expression screening was based on two criteria: the relative expression quantitative Ratio value of the protein and the corresponding T-test P-value. Generally, the Ratio screening condition was that the change factor was greater than 1.5 (when the overall change factor of the protein was large, a 2-fold change factor can be used as the screening condition. When the overall change factor of the protein was small, a 1.3 or 1.2 can be used as the screening condition), and the T-test P value usually used 0.05.

### Molecular docking

The online molecular docking tool HDOCK (http://hdock.phys.hust.edu.cn/) was utilized to predict the binding sites between VHL and ASNS proteins. A more negative docking score suggests a more probable binding model, while a confidence score above 0.7 indicates a likely interaction between the two molecules.

### Tandem Mass Tags (TMT)

The TMT quantitative proteomics analysis and differential protein assessment of ASNS-overexpressing and control Caki-1 cells were conducted by Shanghai Baiqu Biomedical Technology Co., Ltd. Detailed experimental procedures can be found in a previously published article 18. We used the Student's t-test statistical methods to screen for differentially expressed proteins, and the criteria for the differentially expressed proteins were: Q-value < 0.05 and fold change ≤ 0.83 (down-regulated) or ≥ 1.2 (up-regulated).

### Immunofluorescence staining

After dewaxing, paraffin sections were treated with hydrogen peroxide to inhibit endogenous peroxidase activity and repair antigens. Subsequently, these sections were blocked with serum and incubated with the anti-ASNS antibody (CST, 92479, 1:200) overnight at 4°C. Following this, the sections were washed three times with PBS, then incubated with a green fluorescent rabbit secondary antibody for 2 hours at room temperature. After another three washes, the sections were blocked again with serum and incubated with the anti-JUP antibody (Proteintech, 11146-1-AP, 1:200) overnight at room temperature. Following three additional washes, the sections were incubated with a red fluorescent rabbit secondary antibody for 2 hours at room temperature. Finally, the sections were re-stained with the DAPI staining solution and images were captured using a confocal laser microscope to clearly determine the subcellular localization of ASNS and JUP proteins.

### RNA sequencing (RNA-seq)

786-O is the most commonly used RCC cell line with VHL mutations, and the corresponding antibodies have verified the unaccessibility of VHL protein in 786-O cells (CST, 68547). Thus, we conducted RNA-seq on 786-O cells with JUP overexpression and their control cells. Differential expression analysis was performed using the DESeq2 method, followed by KEGG (Kyoto Encyclopedia of Genes and Genomes) pathway enrichment analysis of these genes. The basic function “phyper” in R language was used to calculate the Pvalue, then multiple test correction was performed on these P-value, and finally the screening threshold was set as the Q-value <= 0.05, and log_2_(fold change) ≤ -0.38 (down-regulated) or log_2_(fold change) ≥ 0.38 (up-regulated).

### EdU and TUNEL staining

Apoptotic cells and proliferating cells in the paraffin sections of mouse renal tumors were detected using the EdU and TUNEL DAB staining methods respectively. All procedures were performed strictly according to the manufacturer's instructions (Beyotime, C0085S and C1091).

### Animal experiments

A total of forty five-week-old B-NDG severely immunodeficient mice were randomly assigned to eight groups, with five mice in each group. No blinding was done for animal studies. To establish the orthotopic tumor model in mice, we injected 20μl of the PBS cell suspension containing approximately 10×10^5 live Caki-1 cells or ASNS-overexpressing Caki-1 cells or ASNS-knockdown Caki-1 cells into the subcapsular space of the right kidney of each mouse. One week later, the formation of mouse kidney tumors was observed through *in vivo* imaging of the mice using bioluminescence imaging. Bioluminescence imaging was conducted following previously established methods 18. Two weeks later, for L-Asn-deficient group, L-ASNase (1000 or 5000 IU/kg, Intraperitoneal injection, Once a day for 30 consecutive days) was used; for L-Asn-rich group, L-Asn (3g L-Asn/kg feed ) was used; for ASNS inhibitor group, Bisabosqual A (50-250 IU/kg, Intraperitoneally, daily, for 30 days). Then, bioluminescence imaging was performed on each mice one month later. EdU (50 mg/kg) was administered via intraperitoneal injection two to four hours prior to the euthanasia of the mice. Immediately after euthanasia, *in vitro* imaging of the lungs was performed to assess tumor metastasis. Euthanasia of all mice was performed using carbon dioxide. All procedures were approved by the Institutional Animal Protection and Use Committee of the First Affiliated Hospital, Zhejiang University School of Medicine.

### Statistical analysis

To analyze the differences in the expressions of L-Asn, L-ASNase, ASNS, LysoPA, Indoxyl sulfate, L-Malic acid, Ethyl glucuronide, JUP, pERK1/2, pPI3K, pAKT, Cleaved-Caspase 3, Bcl-2, BAX, E-cadherin, N-cadherin, and SNAIL between two groups, paired or unpaired Student's t-test and the non-parametric Mann-Whitney test were performed using the GraphPad Prism 6 tool. The differences in the Kaplan-Meier survival curve of RCC patients between ASNS and JUP high expression groups and low expression groups were examined through log-rank test using the R language tool. The correlation between JUP expression and VHL and ASNS expression was assessed through linear regression analysis, and the correlation coefficient was analyzed using the Pearson method. All statistical tests were two-sided, and a P value of less than 0.05 was deemed statistically significant.

## Results

### L-Asparagine was identified as a specific differential metabolite in *VHL-*mutant RCC

**Figure 1:**
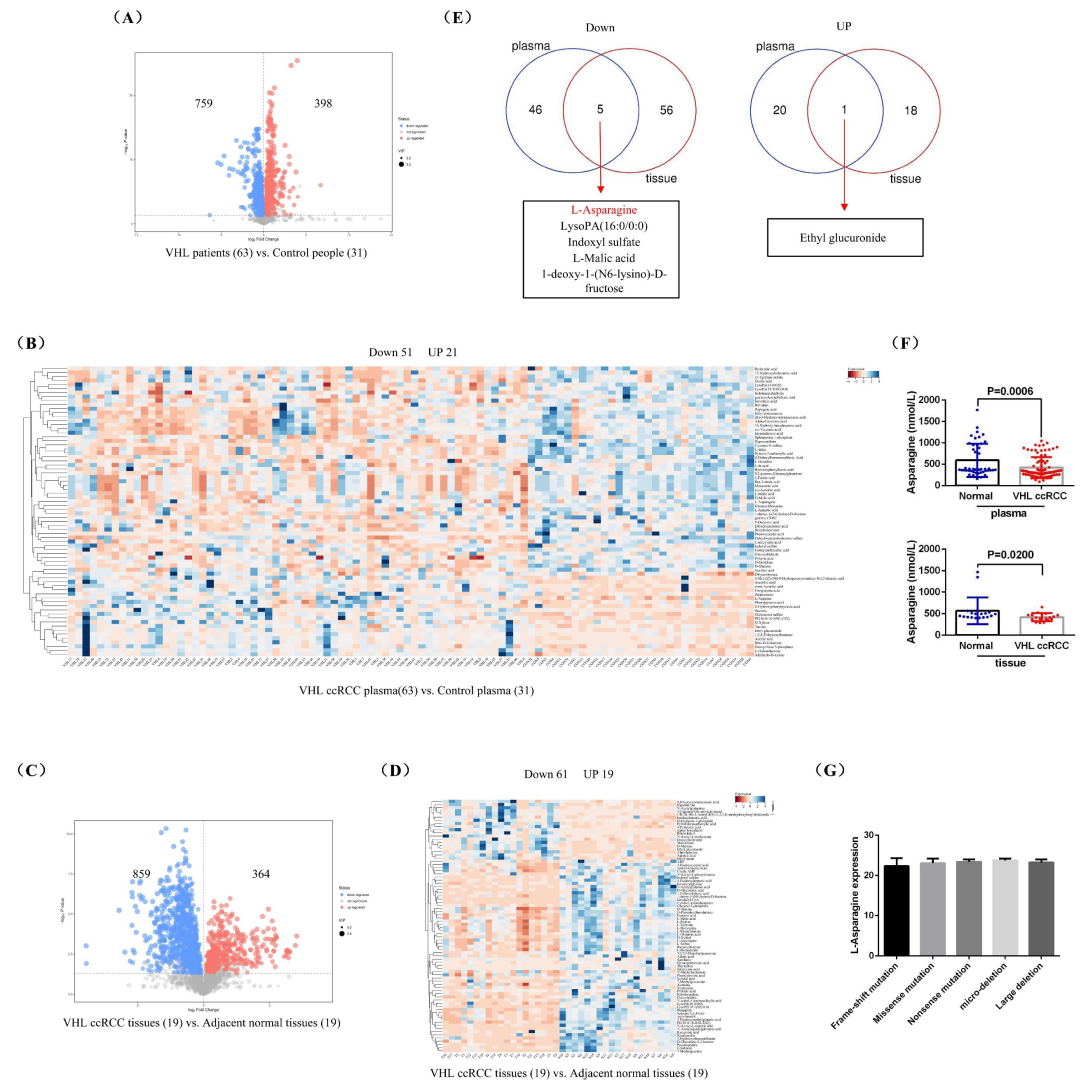
** L-Asparagine was notably underexpressed in *VHL-*mutant RCC.** (A) A volcano plot illustrating the differential metabolites identified through untargeted metabolomics in the plasma of patients with *VHL-*mutant RCC. (B) Hierarchical clustering analysis of the metabolites that were differentially expressed in plasma. (C) A volcano plot showing differential metabolites in untargeted metabolomics of *VHL-*mutant RCC tumor tissues. (D) Hierarchical clustering analysis of differentially expressed metabolites in tumor tissues. (E) A combined analysis of the hierarchical clustering results from both plasma and tumor tissues. (F) ELISA assay measuring the levels of L-Asparagine in plasma and tumor tissues. (G) Comparison of the expression level of L-Asparagine in the plasma of patients with different types of *VHL* gene mutations.

To investigate the differential metabolite in the blood of patients with *VHL-*mutant RCC, we collected plasma samples from 63 *VHL-*mutant RCC patients and 31 healthy control individuals for untargeted metabolomics analysis. The findings revealed that, compared to healthy control individuals, *VHL-*mutant RCC patients exhibited elevated levels of 398 metabolites and reduced levels of 759 metabolites in their plasma (Fig.1A). Hierarchical clustering analysis of these 1157 differentially expressed metabolites identified 21 up-regulated and 51 down-regulated metabolites (Fig.1B). We also performed untargeted metabolomics analysis on 19 pairs of fresh *VHL-*mutant RCC tissues and their adjacent normal (AN) tissues, which showed that 364 metabolites were increased and 859 were decreased in *VHL-*mutant RCC tissues compared to AN tissues (Fig.1C). The hierarchical clustering of these 1223 metabolites resulted in the identification of 19 up-regulated and 61 down-regulated metabolites (Fig.1D). By combining the analysis of differential metabolites from both plasma and tumor tissue samples of *VHL-*mutant RCC patients, we found that 5 metabolites (L-Asparagine, LysoPA(16:0/0:0), Indoxyl sulfate, L-Malic acid, and 1-deoxy-1-(N6-lysino)-D-fructose) were significantly down-regulated in both sample types, while 1 metabolite (Ethyl glucuronide) was significantly up-regulated (Fig.1E). Finally, we validated the expression levels of 5 of these 6 differential metabolites in the plasma of 86 *VHL-*mutant RCC patients and 46 healthy control individuals, as well as in 18 pairs of *VHL-*mutant RCC tissues and their AN tissues, using the ELISA method. Since there was currently no suitable ELISA test kit available, we were unable to verify the expression of 1-deoxy-1-(N6-lysino)-D-fructose this time. Our results confirmed that the levels of L-Asparagine in the plasma and tumor tissue of *VHL-*mutant RCC patients were significantly lower than those in healthy control individuals, while the expressions of LysoPA, Indoxyl sulfate, L-Malic acid, and Ethyl glucuronide showed no significant differences between *VHL*-mutant RCC patients and healthy control individuals, as well as between *VHL*-mutant RCC tissues and their AN tissues (Fig.1F and Supplemental Fig.1). In addition, we compared the expression of L-Asparagine in the plasma of patients with different types of *VHL* gene mutations (frame-shift mutation, missense mutation, nonsense mutation, micro-deletion, and large deletion). The results showed that there was no significant difference in L-asparagine expression among the different groups (Fig.1G). All in all, our findings suggested that L-Asparagine may serve as a specific differential metabolite for patients with *VHL-*mutant RCC.

### Asparagine enhanced the growth and metastasis of RCC *in vitro* and *in vivo*

**Figure 2:**
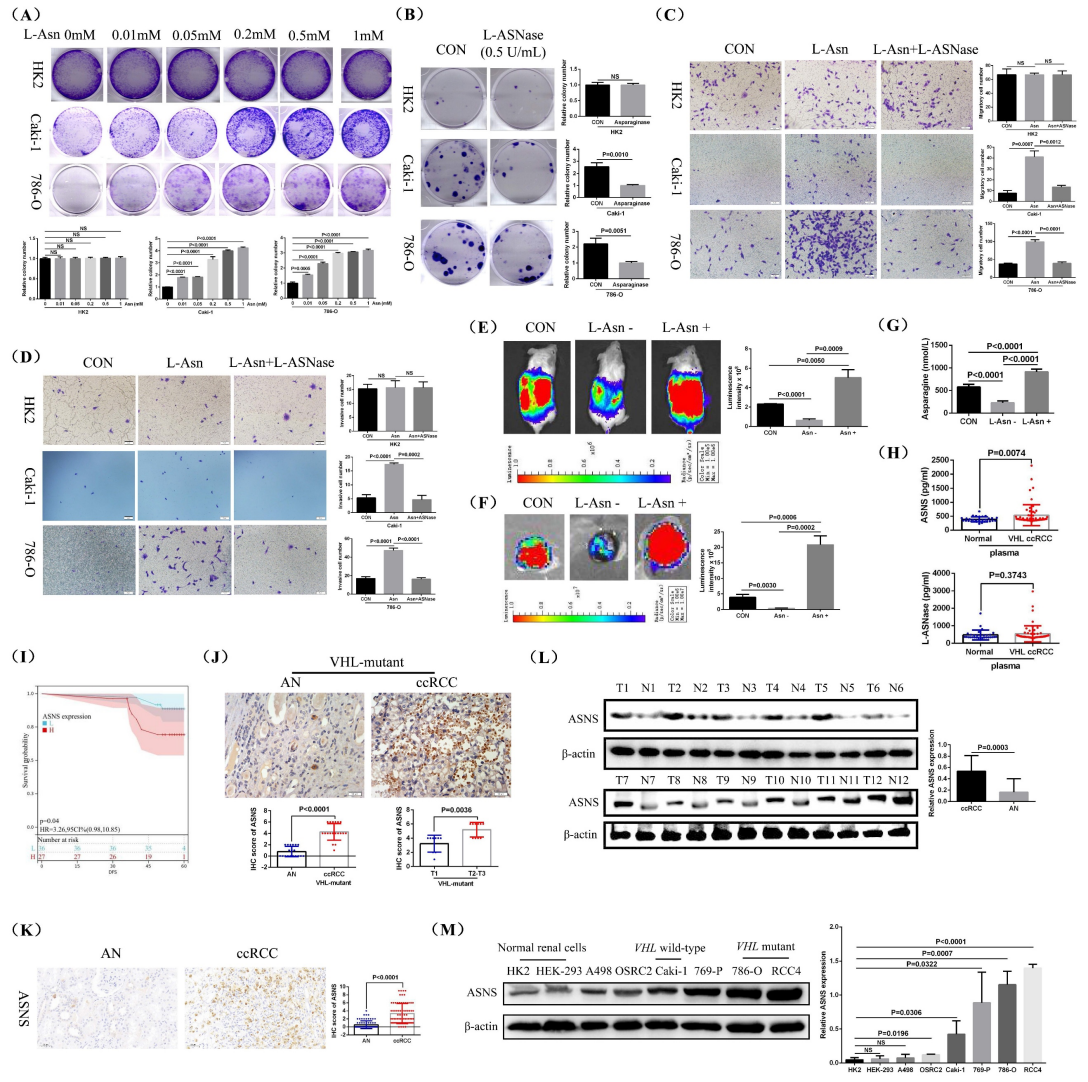
** L-Asparagine enhanced the growth and metastasis of RCC *in vitro* and *in vivo*.** Cell plate cloning experiments assessing HK2, Caki-1 and 786-O cell lines treated with varying concentrations of L-Asparagine (L-Asn). (B) Cell plate cloning experiments evaluating HK2, Caki-1, and 786-O cell lines treated with L-ASNase (0.5 U/mL). (C and D) Experiments measuring cell migration and invasion in HK2, Caki-1, and 786-O cell lines treated with either L-Asn (0.5mM) or L-ASNase (0.5 U/mL). (E) *In vivo* imaging of mouse orthotopic renal tumors fed with either L-Asn-rich (3g L-Asn/kg feed) or deficient diets (Each group consists of 5 mice). (F) *In vitro* imaging of mouse lungs subjected to L-Asn-rich (3g/kg) or -deficient diets (Each group consists of 5 mice). Lung metastasis rate: CON: 5/5; L-Asn -: 4/5; L-Asn +: 5/5. (G) ELISA assay quantifying the levels of L-Asparagine synthase (ASNS) and L-ASNase in mouse kidney tumors. (H) ELISA assay quantifying the levels of ASNS and L-ASNase in plasma. (I) The relationship between the expression of ASNS in the plasma of these 63 *VHL*-mutant RCC patients and the disease-free survival (DFS) of patients. (J) The expression of ASNS in these 19 pairs of *VHL*-mutant RCC and their AN tissues was detected through immunohistochemical staining. (K) Immunohistochemical staining to detect ASNS protein in ccRCC tissues. (L) Western blotting analysis of ASNS protein in RCC tissues. (M) Western blotting analysis of ASNS protein in various RCC cell lines (A498, OSRC2, Caki-1, 769-P, 786-O, and RCC4).

To investigate the biological role of L-Asparagine (L-Asn) in RCC, we supplemented the cell culture medium with different concentrations of L-Asn and monitored the growth of HK2, Caki-1, and 786-O RCC cell lines through cell plate cloning experiments. The findings indicated that the growth rates of both Caki-1 and 786-O cell lines increased significantly with the addition of L-Asn, while the growth rate of the control cell line HK2 showed no significant change (Fig.2A). After removing L-Asn using L-Asparaginase (L-ASNase), the growth rates of Caki-1 and 786-O cell lines were significantly decreased, while the growth rate of HK2 cells remained unchanged (Fig.2B). Additionally, experiments on cell migration and invasion demonstrated that L-Asn enhanced the migratory and invasive capabilities of Caki-1 and 786-O cell lines, whereas the removal of L-Asn significantly hindered these abilities. However, neither L-Asn nor L-ASNase treatment has a significant effect on the migration and invasion abilities of HK2 cells (Fig.2C and D).

Furthermore, animal studies revealed that L-Asn significantly stimulated the growth and spontaneous lung metastasis of orthotopic renal tumors in mice, while reducing L-Asn levels markedly suppressed both growth and spontaneous lung metastasis (Fig.2E and F, Supplemental Fig.2). Furthermore, we measured the content of L-Asn in the renal tumors of mice. The results also demonstrated that L-Asn content was significantly reduced in the L-Asn-deficient group, while it was significantly increased in the L-Asn-rich group (Fig.2G). Therefore, our research results indicated that the growth and metastasis of RCC cells were dependent on the level of L-Asn, and L-Asn can significantly promote the progression of RCC.

Then, we measured the levels of L-Asparagine synthase (ASNS) and L-ASNase in the plasma of patients with *VHL-*mutant RCC and healthy control individuals, discovering that ASNS levels were significantly elevated in RCC patients, while L-ASNase levels remained relatively unchanged (Fig.2H). Based on the survival information of these 63 *VHL-*mutant RCC patients, prognostic analysis revealed that the group with low expression of ASNS in plasma had better DFS (Disease Free Survival) than the group with high expression of ASNS (Fig.2I). Besides, we employed immunohistochemical staining to examine and comparatively analyze ASNS expression in these 19 pairs of *VHL-*mutant RCC tissues and their AN tissues. The results indicated that ASNS expression was significantly higher in RCC tissues compared to the AN tissues, and high ASNS expression correlated with advanced tumor stage (Fig.2J). Additionally, we analyzed ASNS mRNA expression in 539 clear cell RCC (ccRCC) samples and 72 AN tissues using TCGA-KIRC data. Our findings revealed that ASNS expression was notably higher in ccRCC compared to AN tissue. Furthermore, tumors with advanced staging and grading, lymph node invasion, and distant metastasis (T3/T4, G3/G4, Stage III/IV, N1, and M1) exhibited significantly greater ASNS expression than those with lower staging and grading, no lymph node invasion, and no distant metastasis (T1/T2, G1/G2, Stage I/II, N0, and M0) (Supplemental Fig.3A). Prognostic analysis based on patient survival data indicated that patients with high ASNS expression had a significantly lower survival rate compared to those with low ASNS expression (Supplemental Fig.3B). A separate analysis of metastatic ccRCC patients also showed that high ASNS expression correlated with poorer survival outcomes (Supplemental Fig.3C). Then, we compared the expression of ASNS mRNA in *VHL*-mutant ccRCCs and *VHL* wild-type ccRCCs. The analysis results indicated that compared with *VHL* wild-type ccRCCs, the expression of ASNS mRNA showed no significant change in *VHL*-mutant ccRCCs (Supplemental Fig.3D). The prognostic analysis of *VHL*-mutant ccRCC patients also indicated that compared with ASNS low expression group, patients in the high ASNS expression group exhibited shorter overall survival (OS) and DFS (Supplemental Fig.3E).

In order to further explore the expression of ASNS in other RCC pathological types (pRCC and chRCC), we analyzed ASNS expression in tumor and their AN tissues using data from the TCGA-KIRP and TCGA-KICH cohorts, and investigated its correlation with tumor stage and patient prognosis. Our analysis further revealed that ASNS was significantly overexpressed in both pRCC and chRCC. This high expression was closely associated with advanced tumor stage, lymph node invasion, distant metastasis, and adverse clinicopathological features, including poorer OS and DFS (Supplemental Fig.4A and B).

Finally, we confirmed the elevated ASNS protein levels in RCC through immunohistochemical staining and western blotting of clinical ccRCC samples from our center (Fig.2K and L). Additionally, ASNS protein expression was significantly higher in RCC cell lines (A498, OSRC2, Caki-1, 769-P, 786-O, and RCC4) compared to the normal control cell line HK2, particularly in *VHL-*mutant cell lines 786-O and RCC4 (Fig.2M, Supplemental Fig.5).

### VHL regulated ASNS protein expression

**Figure 3:**
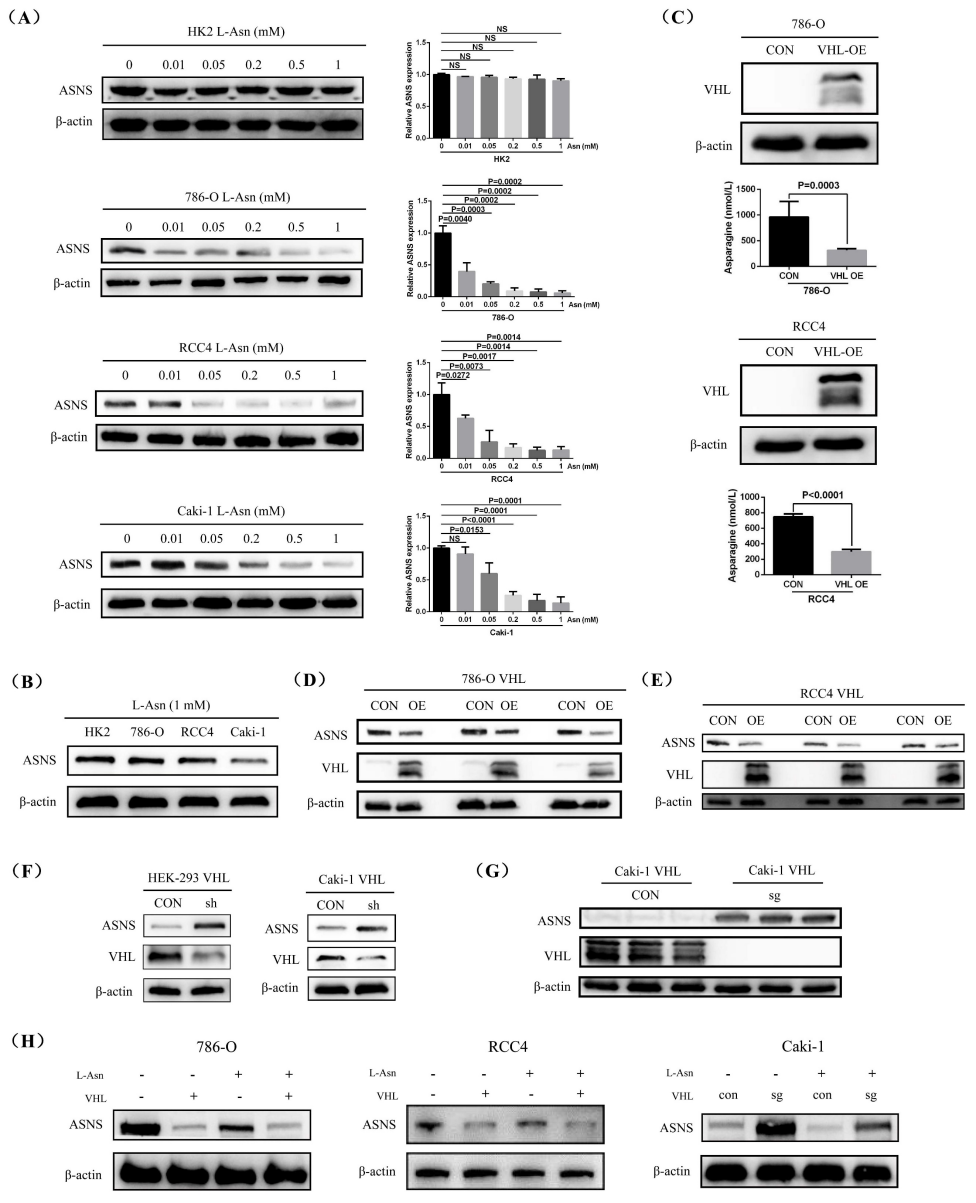
** VHL interacted with ASNS protein and modulated its expression.** (A) Western blotting analysis showing changes in ASNS protein expression in HK2, 786-O, RCC4, and Caki-1 cell lines after treatment with different concentrations of L-Asn. (B) Comparison of ASNS protein expression in HK2, 786-O, RCC4, and Caki-1 cell lines post L-Asn treatment. (C) ELISA analysis of L-Asn expression levels in 786-O and RCC4 cell lines following VHL overexpression. (D and E) Western blotting analysis of ASNS expression changes in 786-O and RCC4 cell lines after VHL overexpression. (F) Western blotting analysis of ASNS expression changes in HEK-293 and Caki-1 cell lines after VHL knockdown. (G) Western blotting analysis of ASNS expression levels in Caki-1 cells after VHL knockout. (H) Confirmation of the regulatory effects of L-Asn and VHL on ASNS expression in 786-O, RCC4, and Caki-1 cell lines.

To further investigate the connection between reduced L-Asn level and increased ASNS protein expression in RCC, we treated HK2, 786-O, RCC4, and Caki-1 cell lines with varying concentrations of L-Asn and measured ASNS protein level. The western blotting results indicated that ASNS protein expression significantly decreased in 786-O, RCC4, and Caki-1 cell lines when sufficient L-Asn was added, while HK2 cells showed no notable change in ASNS expression. Among them, the 786-O cell line might be more sensitive to L-Asn (Fig.3A, Supplemental Fig.6). Furthermore, after adding sufficient L-Asn, the expression of ASNS in Caki-1 cells was still lower compared to that in 786-O and RCC4 cell lines (Fig.3B). Therefore, our findings demonstrated that the low content of L-Asn may promote the high expression of ASNS in RCC.

To further investigate the relationship between VHL expression and L-Asn or ASNS, we first overexpressed VHL in 786-O and RCC4 cell lines and subsequently measured intracellular L-Asn levels. ELISA results showed that VHL overexpression significantly reduced L-Asn levels in both 786-O and RCC4 cells (Fig.3C). Concomitantly, western blotting confirmed that VHL overexpression markedly down-regulated ASNS protein levels in these cell lines (Fig.3D and E), although ASNS mRNA levels remained largely unchanged (Supplementary Fig.7). Additionally, knockdown of VHL in HEK-293 and Caki-1 cells led to a significant increase in ASNS protein expression (Fig.3F). The effect was even more pronounced upon complete knockout of VHL in Caki-1 cells, resulting in a more substantial up-regulation of ASNS (Fig.3G). Finally, we further validated the suppressive effects of L-Asn and VHL on ASNS protein expression in 786-O, RCC4, and Caki-1 cell lines (Fig.3H).

### VHL promoted the ubiquitination of ASNS to suppress its protein expression

**Figure 4:**
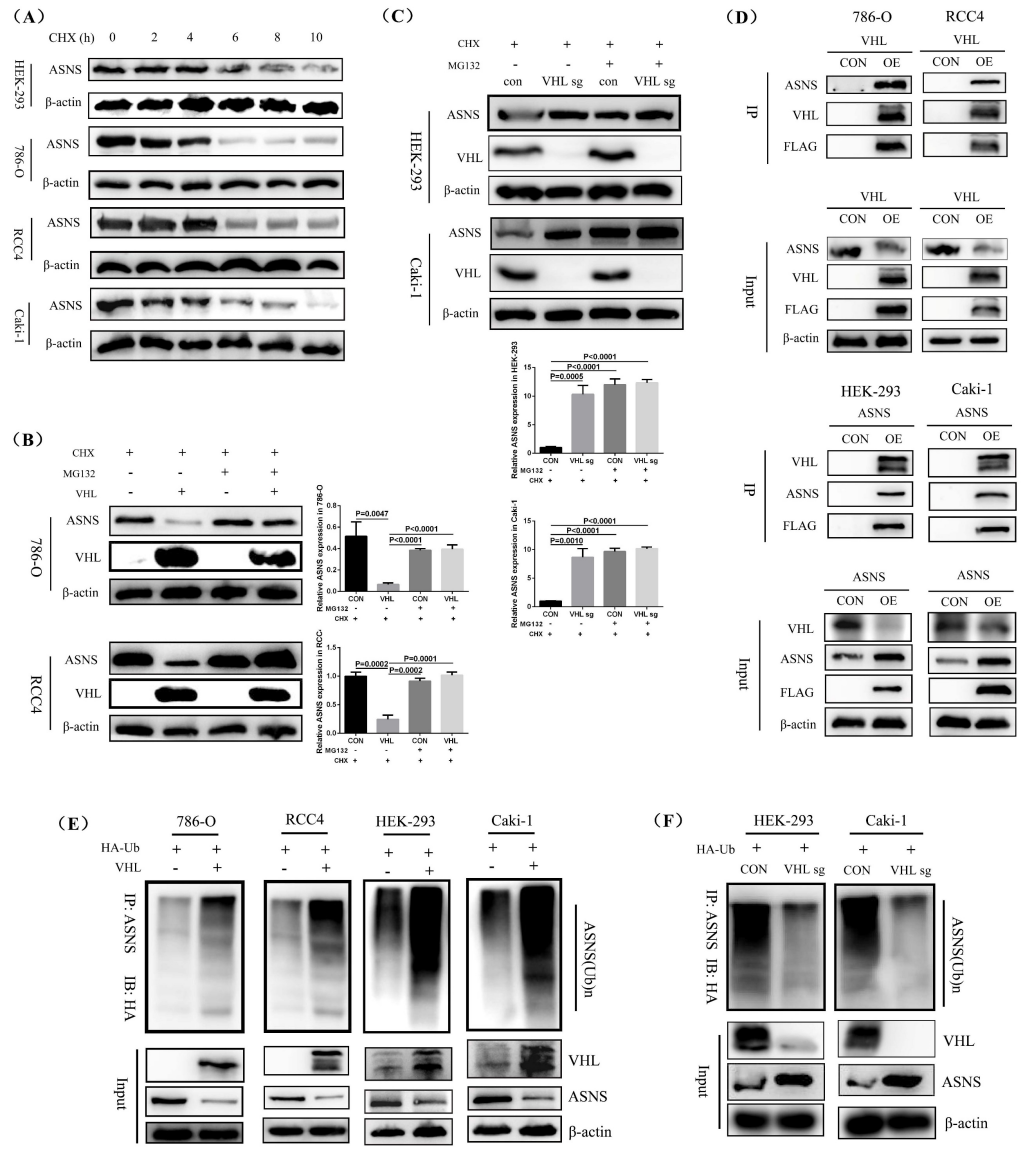
** VHL facilitated the ubiquitination of ASNS and reduced its protein level.** Western blotting analysis of protein stability in HER-293, 786-O, RCC4, and Caki-1 cell lines. (B) Western blotting analysis showing the impact of VHL overexpression on ASNS expression changes in 786-O and RCC4 cell lines following MG132 treatment. (C) Western blotting analysis showing the impact of VHL knockout on ASNS expression changes in HEK-293 and Caki-1 cell lines following MG132 treatment. (D) Confirmation of the interaction between VHL and ASNS proteins in 786-O, RCC4, HEK-293, and Caki-1 cell lines through immunoprecipitation (IP) experiments. (E) Validation of the role of VHL in regulating ASNS protein ubiquitination in 786-O, RCC4, HEK-293, and Caki-1 cell lines using ubiquitination IP experiments. (F) The effect of VHL knockout on ASNS protein ubiquitination using ubiquitination IP experiments in HEK-293 and Caki-1 cell lines.

To investigate how VHL influenced ASNS expression, we examined the effects of CHX (Cycloheximide) on ASNS protein level in HEK-293, 786-O, RCC4, and Caki-1 cell lines at various time intervals. We observed a notable decrease in ASNS protein expression in all these four cell lines after 6 hours of CHX treatment (Fig.4A). Additionally, we treated these cells with MG132 to block proteasome activity and monitored ASNS protein level.

The results showed that after treatment with MG132, there was no significant difference in ASNS expression between VHL-overexpressing 786-O and RCC4 cell lines and their control 786-O and RCC4 cell lines. However, compared to VHL-overexpressing 786-O and RCC4 cell lines not treated with MG132, ASNS protein levels were significantly higher in VHL-overexpressing 786-O and RCC4 cell lines treated with MG132 (Fig.4B, Supplemental Fig.8A). In contrast, compared to VHL-knockout Caki-1 cells untreated with MG132, there was no significant change in ASNS expression in VHL-knockout Caki-1 cells treated with MG132 (Fig.4C, Supplemental Fig.8B). This indicated that VHL overexpression influenced the proteasomal degradation pathway of ASNS protein. Furthermore, we conducted immunoprecipitation (IP) assays to assess the interaction between VHL and ASNS protein, which confirmed a strong binding affinity of VHL to ASNS in the 786-O, RCC4, HEK-293, and Caki-1 cell lines (Fig.4D). Additionally, our *in vitro* ubiquitination IP experiments revealed that the ubiquitination levels of ASNS protein were significantly elevated in VHL-overexpressing 786-O, RCC4, HEK-293, and Caki-1 cell lines compared to their control 786-O, RCC4, HEK-293, and Caki-1 cell lines (Fig.4E). Further, we conducted ubiquitination IP experiments in VHL-knockout HEK-293 and Caki-1 cells. The results showed that, compared with the control HEK-293 and Caki-1 cell lines, the ubiquitination level of the ASNS protein was significantly reduced in VHL-knockout HEK-293 and Caki-1 cell lines (Fig.4F). Thus, our findings suggest that VHL may reduce ASNS protein expression by enhancing its ubiquitination.

### VHL ubiquitinated the 510 lysine residue of the ASNS protein to modulate its expression

**Figure 5:**
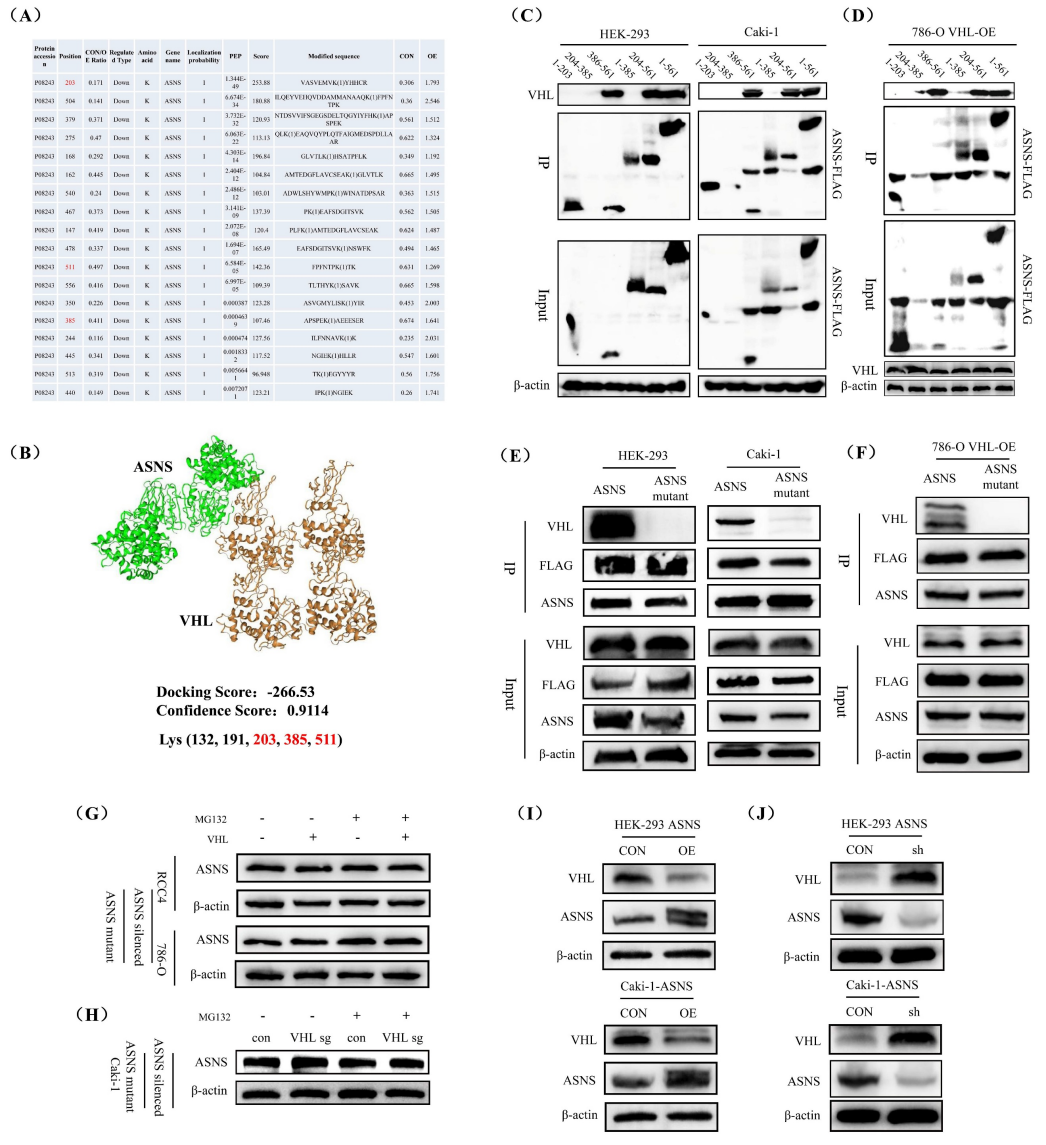
** VHL ubiquitinated the 510 lysine residue of ASNS protein to modulate its expression.** Identification of distinct ubiquitination modification sites on ASNS protein in VHL-overexpressing 786-O cells compared to control cells through ubiquitination proteomic analysis. (B) Prediction of the binding site between VHL and ASNS proteins using the online docking tool HDOCK. (C) Assessment of the binding of VHL to five truncated ASNS proteins via IP experiments in HEK-293 and Caki-1 cell lines. (D) Assessment of the binding of VHL to five truncated ASNS proteins via IP experiments in VHL overexpressed 786-O cells. (E) Examination of the interaction between VHL and ASNS mutants in HEK-293 and Caki-1 cell lines. (F) Examination of the interaction between VHL and ASNS mutants in VHL overexpressed 786-O cells. (G) Western blotting analysis of the effects of VHL overexpression and MG132 treatments on ASNS protein levels in ASNS mutant RCC4 and 786-O cell lines. (H) Western blotting analysis of the effects of VHL knockout and MG132 treatments on ASNS protein levels in ASNS mutant Caki-1 cells. (I) Evaluation of changes in VHL expression in HEK-293 and Caki-1 cell lines following ASNS overexpression. (J) Assessment of VHL expression changes in HEK-293 and Caki-1 cell lines after ASNS knockdown.

To identify the specific ubiquitination sites of VHL on the ASNS protein, we performed an initial proteomic analysis of ubiquitination modifications in 786-O cells overexpressing VHL, compared to control 786-O cells. The results revealed that the ubiquitination levels of 18 ASNS protein sites (lysine residues 203, 504, 379, 275, 168, 162, 540, 467, 147, 478, 511, 556, 350, 385, 244, 445, 513, 440) were significantly higher in the VHL-overexpressing cells (Fig.5A). We then utilized the online molecular docking tool HDOCK to predict potential binding sites between VHL and ASNS proteins, which suggested that VHL could bind to ASNS at five lysine residue sites (positions 132, 191, 203, 385, 511) (Fig.5B). Based on the findings from the ubiquitination proteomic analysis and molecular docking prediction, we hypothesized that VHL may regulate ASNS protein expression by influencing the ubiquitination levels of lysine residues at positions 203, 385, and 511. To explore these potential binding sites, we created five truncated versions of the ASNS protein (amino acids 1-203, 204-385, 386-561, 1-385, 204-561). Using these truncated ASNS proteins for IP experiments, we determined that the binding region between VHL and ASNS was located between amino acids 386-561, with the 510 lysine residue identified as the likely modification site in HEK-293 and Caki-1 cell lines (Fig.5C). Furthermore, we also conducted IP experiments of these five truncated ASNS proteins in VHL-overexpressing 786-O cells, and obtained results that were consistent with those obtained in HEK-293 and Caki-1 cell lines (Fig.5D).

To further validate this, we generated a mutant ASNS protein with a change at the 510 lysine position (AAA-AGA, lysine to arginine) and conducted IP experiments. The results confirmed that mutating the 510 lysine significantly reduced the binding affinity between ASNS and VHL protein in HEK-293 and Caki-1 cell lines (Fig.5E). Moreover, mutating the 510 lysine residues also remarkably reduced the binding affinity of ASNS to VHL protein in VHL-overexpressing 786-O cells (Fig.5F). Additionally, VHL overexpression and treatment with MG132 did not alter ASNS protein levels in RCC4 and 786-O cell lines after the 510 lysine mutation (Fig.5G), and VHL knockout and MG132 treatment also did not alter the ASNS protein level in Caki-1 cell line after the 510 lysine mutation (Fig.5H). Finally, we investigated whether changes in ASNS expression may affect VHL expression, and our results found that ASNS overexpression significantly decreased VHL protein levels, while ASNS knockdown notably increased VHL protein levels in HEK-293 and Caki-1 cell lines (Fig.5I and J).

### ASNS bound to JUP protein and regulated its expression

**Figure 6:**
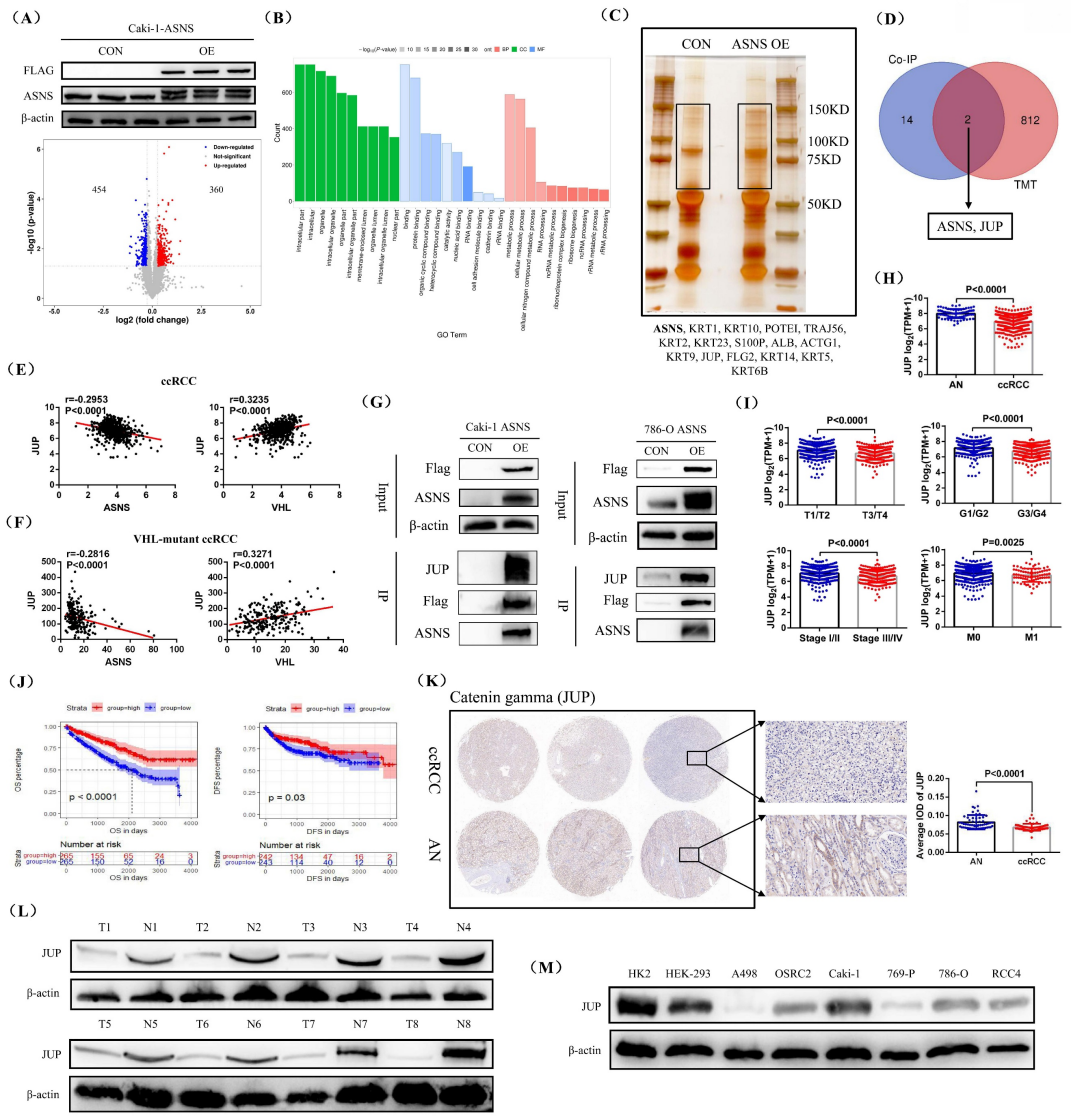
** ASNS interacted with JUP protein and affected its expression.** Molecular volcano plot illustrating differential expression in ASNS-overexpressing Caki-1 cells compared to control Caki-1 cells based on TMT proteomic analysis. (B) GO pathway enrichment analysis for differentially expressed molecules. (C) Silver staining results showing ASNS overexpression and its control in IP samples from Caki-1 cells. (D) Combined analysis of TMT proteomics and mass spectrometry results from silver-stained differential protein bands. (E) Correlation analysis of JUP expression with ASNS and VHL expression using TCGA-KIRC data. (F) Correlation analysis of JUP expression with ASNS and VHL expression using the data from *VHL*-mutant ccRCC tissues in TCGA-KIRC. (G) Confirmation of the interaction between ASNS and JUP proteins in Caki-1 and 786-O cell lines through IP experiments. (H and I) Analysis of JUP expression characteristics in ccRCC and its association with various pathological stages, grades, and distant metastasis using TCGA-KIRC data. (J) Comparison of overall survival (OS) and DFS between patients with high and low JUP expression. (K) Validation of JUP expression in clinical samples from our center using immunohistochemical staining. (L) Western blotting analysis of JUP protein in ccRCC tissues. (M) Western blotting analysis of ASNS protein in various RCC cell lines (A498, OSRC2, Caki-1, 769-P, 786-O, and RCC4).

To investigate the downstream regulatory mechanism of ASNS in RCC cells, we chose to overexpress ASNS in the *VHL* wild-type RCC cell line Caki-1, where the ASNS expression was lower compared to 786-O and RCC4 cell lines. Then, we employed TMT proteomics sequencing to determine the differentially expressed proteins in the ASNS-overexpressing Caki-1 cells compared to the control Caki-1 cells. Our western blotting results showed that ASNS protein was successfully overexpressed in Caki-1 cells, and the analysis results revealed that in ASNS-overexpressing Caki-1 cells, 360 proteins were up-regulated and 454 proteins were down-regulated relative to the control Caki-1 cells (Fig.6A). GO enrichment analysis indicated that these differentially expressed proteins were primarily enriched in pathways related to protein binding and cellular metabolism (Fig.6B). We then performed silver staining to visualize the differential protein expression bands between the IP samples from ASNS-overexpressing Caki-1 cells and the control Caki-1 cells, followed by protein mass spectrometry to identify the proteins in these bands. The final detection results identified 16 potential ASNS-binding proteins (Fig.6C). Finally, by integrating the TMT proteomics data with the mass spectrometry results, we identified JUP (Junction plakoglobin) as a downstream regulatory molecule of ASNS (Fig.6D). Correlation analysis of TCGA-KIRC data revealed that JUP expression was negatively correlated with ASNS but positively correlated with VHL expression (Fig.6E). In addition, we selected ccRCC tissues with *VHL* mutations in TCGA-KIRC and analyzed the correlation between JUP expression and ASNS and VHL expression. The results also confirmed a significant negative correlation between JUP expression and ASNS expression and a significant positive correlation between JUP expression and VHL expression in *VHL*-mutant ccRCC (Fig.6F). We further confirmed the interaction between ASNS and JUP proteins in Caki-1 and 786-O cell lines through IP experiments (Fig.6G). Furthermore, analysis of TCGA-KIRC data showed that JUP expression was significantly down-regulated in ccRCC compared to AN tissues. Reduced JUP expression was associated with advanced tumor stages, grades, and metastasis (T3/T4, G3/G4, Stage III/IV, N1, and M1) (Fig.6H and I). Prognostic analysis revealed that patients with high JUP expression had significantly longer OS and DFS compared to those with low JUP expression (Fig.6J). Finally, we confirmed the low expression of JUP protein in RCC through immunohistochemical staining and western blotting of clinical samples from our center (Fig.6K and L). Similarly, in the RCC cell lines, the protein expression of JUP was also generally reduced (Fig.6M).

### ASNS modulated the PI3K-AKT and MAPK pathways by interacting with JUP

**Figure 7:**
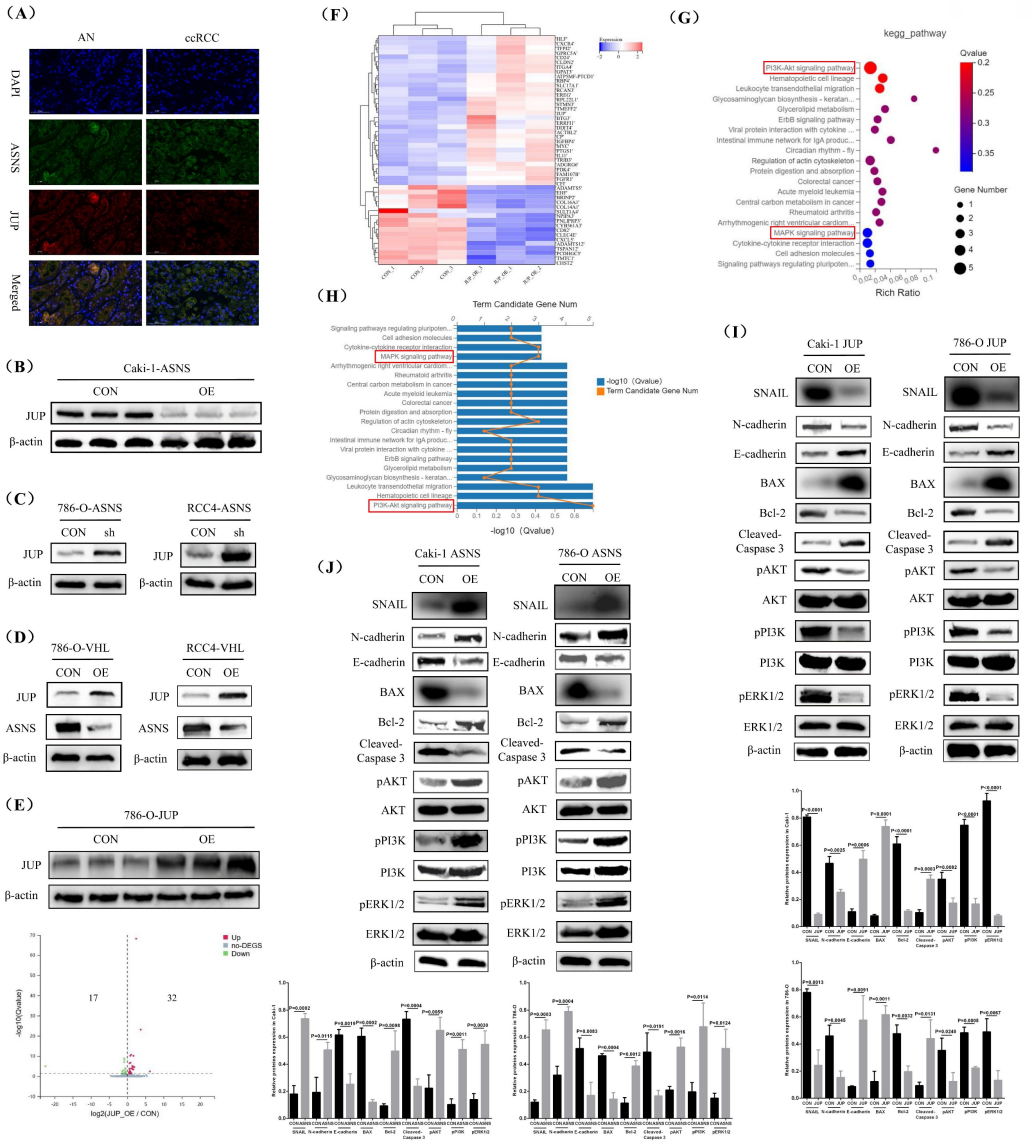
** ASNS modulated the PI3K-AKT and MAPK pathways by interacting with JUP.** Immunofluorescence staining revealed the sublocalization of ASNS and JUP protein expression in RCC tissues. (B) Western blotting analysis reveals changes in JUP protein expression following ASNS overexpression in Caki-1 cells. (C) Western blotting analysis shows alterations in JUP expression after ASNS knockdown in 786-O and RCC4 cell lines. (D) Western blotting analysis indicates changes in JUP expression after ASNS overexpression in 786-O and RCC4 cell lines. (E) A volcano plot illustrates the analysis of differentially expressed genes in JUP-overexpressing 786-O cells compared to control 786-O cells using RNA-seq. (F) The expression heat-map of these 49 differential genes. (G) KEGG pathway enrichment analysis for these differentially expressed genes. (H) Graph showing the number of differentially expressed genes included in the KEGG enrichment pathways. (I) Western blot analysis detects changes in protein expression levels of pERK1/2, pPI3K, pAKT, Cleaved-caspase 3, Bcl-2, BAX, E-cadherin, N-cadherin, and SNAIL following JUP overexpression in Caki-1 and 786-O cell lines. (J) Western blotting analysis shows changes in protein expression levels of pERK1/2, pPI3K, pAKT, Cleaved-Caspase 3, Bcl-2, BAX, E-cadherin, N-cadherin, and SNAIL after ASNS overexpression in Caki-1 and 786-O cell lines.

To better understand the regulatory influence of ASNS on JUP and its downstream pathways, we initially performed immunofluorescence staining to observe the co-localization of ASNS and JUP proteins in RCC, and the test results confirmed that although the expression levels of ASNS and JUP proteins were significantly negatively correlated, there was a clear co-expression phenomenon in ccRCC (Fig.7A). Subsequently, we conducted western blotting analysis to assess JUP protein level in Caki-1 cells with ASNS overexpression compared to control Caki-1 cells, revealing a significant decrease in JUP expression following ASNS overexpression (Fig.7B). Additionally, we also investigated the effect of ASNS knockdown on the expression of JUP in the 786-O and RCC4 cell lines, which showed a notable increase in JUP level after ASNS was knocked down (Fig.7C). Furthermore, we analyzed JUP expression changes following VHL overexpression, finding that JUP levels were significantly elevated in VHL-overexpressing 786-O and RCC4 cell lines compared to the control 786-O and RCC4 cells (Fig.7D).

To further clarify the downstream regulatory pathways of JUP, we chose to overexpress JUP in the *VHL*-mutated RCC line and conduct transcriptome sequencing. Firstly, our western blotting test results confirmed the effectiveness of JUP overexpression. Then, we utilized RNA-seq to identify differentially expressed genes in JUP-overexpressing 786-O cells versus control 786-O cells, which revealed 32 significantly up-regulated genes and 17 down-regulated genes in the JUP-overexpressing cells (Fig.7E). The names of these 49 genes and their expression status were shown in Fig.7F. KEGG pathway enrichment analysis indicated that alterations in JUP expression were closely associated with the PI3K-AKT and MAPK pathways (Fig.7G). 5 genes (MYC, EREG, FGFR1, DDIT4, and ITGA4) were involved in the PI3K-AKT pathway and 3 genes (MYC, EREG, and FGFR1) were involved in the MAPK pathway (Fig.7H). Finally, we performed western blotting to evaluate the expression of key molecules in these two pathways (pERK1/2, pPI3K, and pAKT). The results demonstrated that JUP overexpression significantly inhibited the PI3K-AKT and MAPK pathways while enhancing the expression of apoptosis and metastasis inhibition related proteins (Cleaved-Caspase 3, BAX, and E-cadherin) (Fig.7I, Supplemental Fig.9). In contrast, ASNS overexpression notably activated the PI3K-AKT and MAPK pathways and increased the levels of anti-apoptotic and metastasis-promoting proteins (Bcl-2, N-cadherin, and SNAIL) (Fig.7J, Supplemental Fig.10).

### Knocking down ASNS and utilizing ASNS inhibitor significantly restrained the growth and metastasis of RCC *in vitro* and *in vivo*

**Figure 8:**
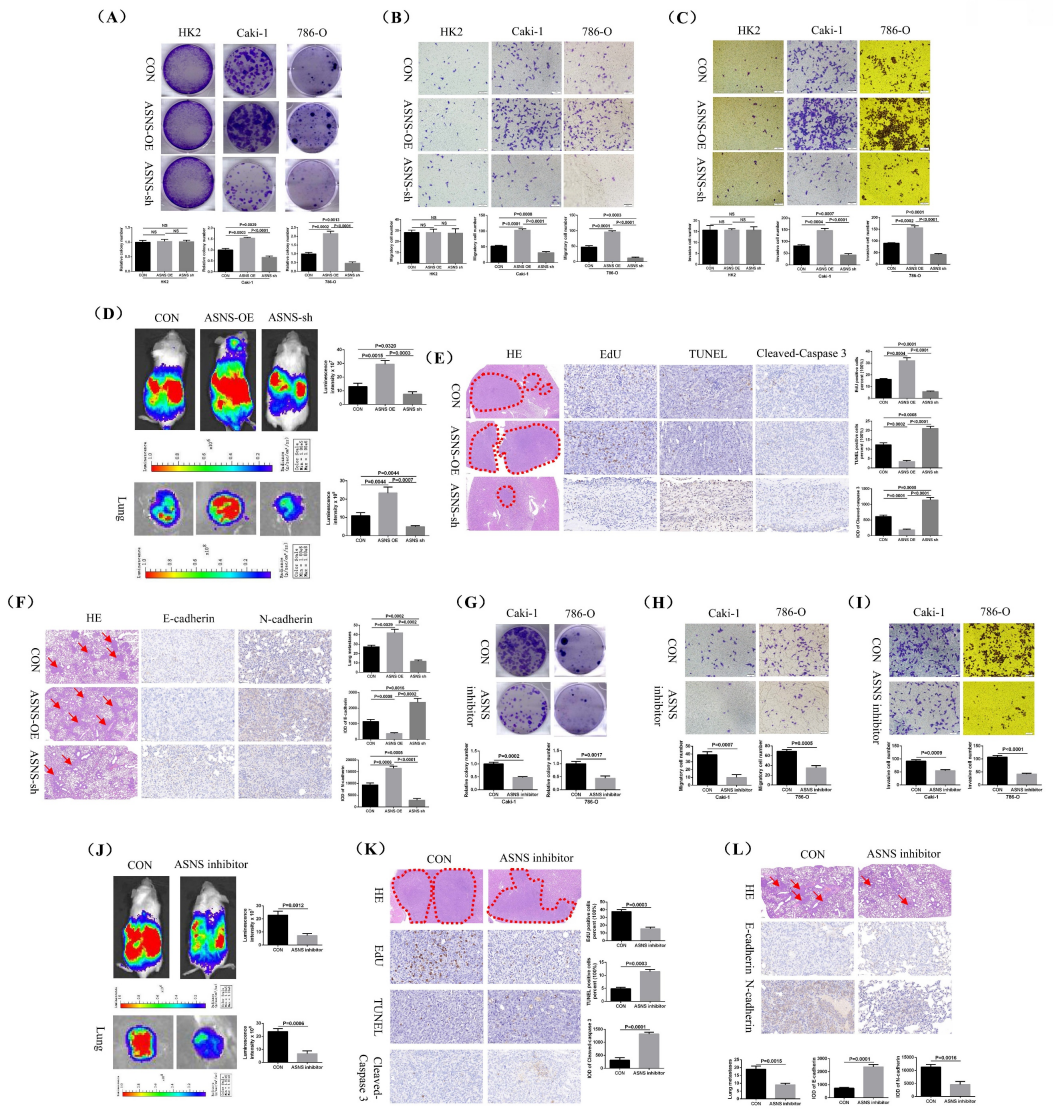
** Silencing ASNS and using an ASNS inhibitor significantly reduced RCC growth and metastasis *in vitro* and* in vivo*.** The impact of ASNS overexpression and knockdown on the growth of HK2, Caki-1 and 786-O cell lines was evaluated using a colony formation assay. (B and C) Transwell migration and invasion assays were performed to assess the effects of ASNS overexpression and knockdown on the migration and invasion capabilities of HK2, Caki-1, and 786-O cell lines. (D) The influence of ASNS overexpression and knockdown on the growth and spontaneous lung metastasis of orthotopic renal tumors in mice was analyzed. (E) The effects of ASNS overexpression and knockdown on EdU, TUNEL, and Cleaved-Caspase 3 expression in mouse renal tumors were examined. (F) The impact of ASNS overexpression and knockdown on E-cadherin and N-cadherin expression in mouse lung metastases was assessed. (G) The effect of the ASNS inhibitor (Bisabosqual A) on the growth of Caki-1 and 786-O cell lines was evaluated through cell plate cloning experiments. (H and I) The influence of ASNS inhibitor on the migration and invasion of Caki-1 and 786-O cell lines was analyzed through migration and invasion assays. (J) The effect of ASNS inhibitor on the growth and spontaneous lung metastasis of mouse renal tumors was assessed. (K) The effects of ASNS inhibitor on EdU, TUNEL, and Cleaved-Caspase 3 expression in mouse renal tumors were examined. (L) The impact of ASNS inhibitor on E-cadherin and N-cadherin expression in mouse lung metastases was analyzed.

To further investigate the role of ASNS in RCC, we performed experiments on cell colony formation, migration, and invasion to assess how changes in ASNS expression affected cell growth and metastatic potential *in vitro*. Our results demonstrated that ASNS overexpression markedly promoted the growth, migration, and invasion capabilities of Caki-1 and 786-O cell lines, whereas knocking down ASNS led to a marked decrease in these abilities. However, the change in ASNS expression had no significant impact on the growth, migration and invasion abilities of the control cell line HK2 (Fig.8A-C). We then employed a mouse orthotopic tumor model to evaluate how alterations in ASNS expression influenced RCC growth and metastasis *in vivo*. The results revealed that mouse renal tumors with ASNS overexpression exhibited accelerated growth and increased spontaneous lung metastasis compared to the control group, while tumors with ASNS knockdown showed significantly reduced growth and metastasis (Fig.8D).

Additionally, we performed immunohistochemical staining to assess the levels of EdU (a marker for cell proliferation), TUNEL (a marker for cell apoptosis), and Cleaved-Caspase 3 in mouse renal tumors. The results confirmed that EdU level was significantly higher and TUNEL and Cleaved-caspase 3 levels were lower in the ASNS overexpression group, while the opposite was true for the ASNS knockdown group (Fig.8E). We also used H&E staining to examine lung metastases and immunohistochemical staining to evaluate E-cadherin and N-cadherin expression in these metastases. The findings showed that renal tumors with ASNS overexpression had significantly more lung metastases, decreased E-cadherin expression, and increased N-cadherin expression compared to the control group, while the ASNS knockdown group exhibited fewer lung metastases, increased E-cadherin, and decreased N-cadherin (Fig.8F).

Moreover, treatment of RCC cells with the ASNS inhibitor Bisabosqual A further confirmed that the growth, migration, and invasion of Caki-1 and 786-O cell lines were significantly inhibited (Fig.8G-I). Animal studies also demonstrated that the ASNS inhibitor effectively suppressed the growth and spontaneous lung metastasis of mouse kidney tumors (Fig.8J). Immunohistochemical analysis of tumor tissues showed that treatment with ASNS inhibitor resulted in a significant reduction in EdU expression and an increase in TUNEL and Cleaved-Caspase 3 expression (Fig.8K). Similarly, H&E staining for lung metastases and immunohistochemical staining for E-cadherin and N-cadherin revealed that after ASNS inhibitor treatment, E-cadherin expression was significantly increased while N-cadherin expression was notably decreased (Fig.8L).

**Figure 9:**
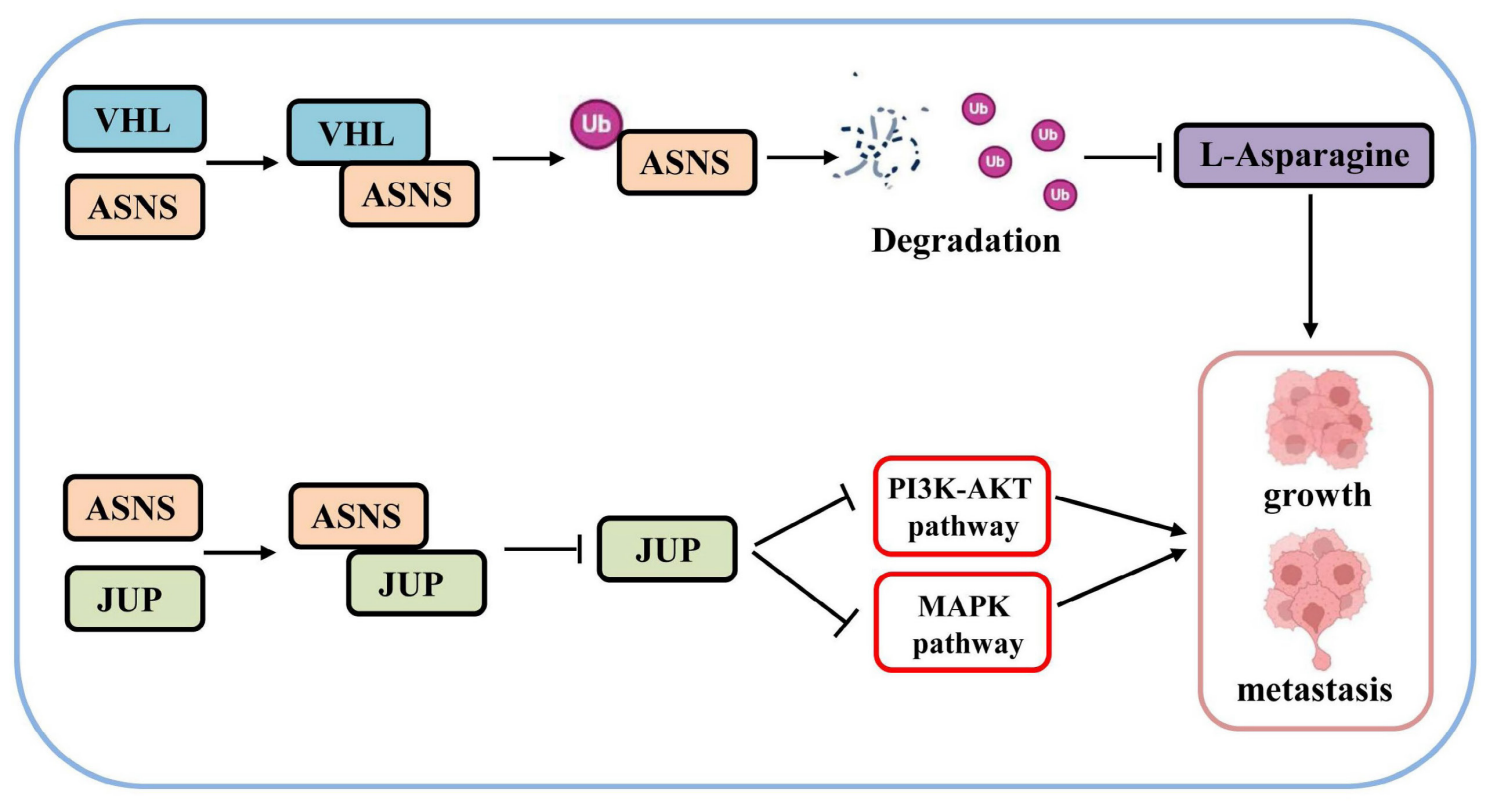
** The mechanism diagram of VHL/ASNS inhibiting the growth and metastasis of RCC**.

Based on these above results, we have identified a novel mechanism through which VHL/ASNS suppresses the growth and metastasis of RCC by modulating JUP and influencing the PI3K-AKT and MAPK pathways (Fig.9).

## Discussion

Cancer cells have the ability to modify their metabolism to fulfill the heightened energy and anabolic requirements necessary for ongoing cell division. Amino acid metabolism plays a crucial role in the metabolic reprogramming of tumors and is a significant factor in tumor growth and malignant advancement. Current therapeutic strategies that target the metabolism of arginine, glycine, and glutamine have demonstrated encouraging anti-cancer effects 19. Furthermore, recent studies have demonstrated that L-Asn plays a pivotal role in mitochondrial respiration, cell survival, and tumor progression 20. L-Asn is classified as a non-essential amino acid. Within the body, ASNS facilitates the conversion of aspartic acid and glutamine into L-Asn and glutamic acid to satisfy the L-Asn needs of tumor cells, while L-ASNase aids in breaking down L-Asn into aspartic acid 21. In most patients with acute lymphoblastic leukemia (ALL), leukemic cells exhibit low levels of ASNS and are dependent on the supply of extracellular L-Asn. Clinically, L-ASNase has been effectively utilized in treating ALL, due to its targeted action and capacity to reduce circulating L-Asn levels 22, and ASNS can serve as a biomarker for ALL patients undergoing L-ASNase therapy 23. However, ALL blasts could synthesize and secrete glutamine, thereby increasing the extracellular glutamine content available to stromal cells and driving human primary bone marrow mesenchymal stromal cells to produce L-Asn, which was then secreted to maintain the survival of L-Asn dependent cancer cells 24. Additionally, recent research has indicated that both supra-physiological and physiological concentrations of L-Asn can inhibit de novo L-Asn synthesis, and overexpression of ASNS in L-ASNase-sensitive B-cell lymphomas (BCLs) *in vitro* was not sufficient to confer resistance to L-ASNase treatment *in vivo* 25. Therefore, the baseline level of ASNS expression (low or high) may not determine the dependence of BCL on de novo L-Asn biosynthesis and cannot predict BCL sensitivity to L-ASNase activity 25. In solid tumors, research has shown that L-Asn can enhance the proliferation or metastasis of prostate, breast, and lung cancers. In gastric and liver cancer cell lines, a vulnerability related to ASNS has been identified, while soft tissue sarcoma, hepatocellular carcinoma, and ovarian cancer exhibit greater sensitivity to L-ASNase 26-28. L-Asn can inhibit IFN-I signal transduction and promote the immune escape of bladder cancer, and the cooperation of L-ASNase and anti-PD-1 therapy will be one of the promising programs to inhibit tumor growth 29.

In our study, we found a notable reduction in L-Asn expression in* VHL-*mutant RCC. However, our *in vitro* experiments demonstrated that adding L-Asn to the cell culture medium significantly increased the growth rate of the 786-O and Caki-1 RCC cell lines, while removing L-Asn with L-ASNase led to a marked inhibition of cell growth. In our mouse model of orthotopic renal tumor growth, the L-Asn-deficient group showed significantly reduced renal tumor growth and spontaneous lung metastasis, whereas the L-Asn-enriched group experienced enhanced tumor growth and metastasis. These findings suggested that L-Asn may contribute to the development of RCC. Furthermore, patients with *VHL*-mutant RCC exhibited significantly higher plasma levels of ASNS enzyme compared to healthy controls, whereas the levels of L-ASNase remained unchanged. Analysis of TCGA-KIRC data, in conjunction with our immunohistochemical staining and western blotting results, consistently confirmed the high expression of ASNS in ccRCC. Thus, although there was a state of low L-Asn expression and high ASNS expression in RCC, the high demand for L-Asn by RCC may lead to the depletion of L-Asn and its low-level state and the promotion of ASNS expression. However, high ASNS expression might also be the result of metabolic reprogramming in RCC, rather than the cause. To answer this question, plasma samples need to be collected from more RCC patients in the near future for metabolomics analysis. Analyzing the changes in the L-Asn metabolic pathway in a large sample will help clarify the specific role of ASNS in RCC.

Although our ELISA experiment results did not confirm that the differential metabolites (LysoPA, Indoxyl sulfate, L-Malic acid, and Ethyl glucuronide) detected by metabolomics, showed differences in expression between *VHL*-mutant RCC and the control group, they may still play a certain role in renal cancer. For example, indoxyl sulfate may act as a bridge between chronic kidney disease and urothelial carcinoma by promoting the uptake and metabolism of glutamine 30; biological synthesis of Poly(β-L-malic acid) (PMLA) can be used as a delivery nanoplatform for transformative cancer medicine 31; alcohol consumption was associated with increased risk of oxidative stress and upper respiratory and digestive tract cancer, and alcohol consumption can be estimated by measuring ethyl glucuronide in hair 32.

When the demand of body for L-Asn is limited, a broad stress response is triggered, which includes reactions to amino acid scarcity and the unfolded protein response. These responses enhance the transcription of ASNS through signaling pathways like GCN2/eIF2/ATF4 and PERK/eIF2/ATF 33, 34. Additionally, in conditions of L-Asn deficiency, the protein ZBTB1 can attach to the ASNS promoter, boosting ASNS transcription^35^. Increasing dietary L-Asn can facilitate tumor metastasis by promoting the production of proteins involved in epithelial-mesenchymal transition, while a decrease in L-Asn availability can inhibit tumor growth by suppressing the mTOR pathway or lowering oncogene expression 27. Furthermore, specific genetic alterations in oncogenes or tumor suppressor genes may render tumor cells vulnerable to selective metabolic changes, leading to distinct metabolic profiles. Previous research has indicated that mutations in tumor suppressor genes can result in resistance to L-ASNase, impacting tumor treatment effectiveness. For instance, studies have shown that mutant TP53 can elevate L-Asn levels in tumor cells by increasing ASNS expression, thus supporting tumor growth and proliferation 36. Recurrent ALL with *TP53* mutations has developed resistance to L-ASNase through ASNS up-regulation. Consequently, APR-246, a drug that targets ASNS for treating hematological cancers with *TP53* mutations, may work synergistically with L-ASNase in recurrent ALL cases with these mutations 37. During this study, we found that after VHL overexpression, L-Asn level in RCC4 and 786-O cell lines was significantly reduced. Besides, ASNS protein level was also remarkably decreased in RCC4 and 786-O cell lines after VHL overexpression, although ASNS mRNA levels did not show significant variation. Mechanistically, our IP experiments and ubiquitination modification proteomics confirmed that VHL protein interacts with ASNS protein, promoting its ubiquitination. However, the specific underlying mechanism remains unclear and requires further investigation. It is also necessary to explore the exact role of *VHL* mutations (different types of *VHL* gene mutations, VHL inactivation or inactive mutations) in regulating ASNS expression, L-Asn level, and the proliferation and metastasis of RCC cells. This is indeed worthy of further exploration. Therefore, we need to collect more samples of *VHL*-mutant RCC to classify the *VHL* mutation types, including frame-shift mutation, missense mutation, nonsense mutation, micro-deletion, and large deletion, and conduct verification in the near future. Furthermore, the interplay between ASNS and HIFs warrants further investigation to validate that ASNS is a novel downstream ubiquitination target of VHL, independent of HIFs.

In addition, our TMT proteomics analysis and IP experiments revealed that ASNS can bind to and hinder the protein expression of Junction plakoglobin (JUP). JUP, as a homolog of β-catenin, is an important cell adhesion molecule that facilitates cell attachment. Cancer is marked by a breakdown in normal cell regulation, resulting in unchecked cell growth and the disruption of cell-to-cell connections, which allows malignant cells to invade nearby tissues. The role of JUP in different malignant tumors may be controversial, depending on its ability to interact with β-catenin as a transcription factor. Recent research has also highlighted the role of JUP role in various cell signaling pathways, such as MAPK and PI3K-AKT, underscoring its crucial role in tumorigenesis, progression, and prognosis 38-41. For instance, the absence of JUP in the cell membrane and/or cytoplasm of gastric cancer cells eliminated the inhibitory effect of JUP on EGFR at the cell membrane, leading to elevated level of p-AKT and enhanced activity of the AKT/GSK3β/β-catenin signaling pathway. In turn, nuclear JUP interacted with nuclear β-catenin and TCF4, and cooperated with β-catenin to promote the transcription of TCF4 and the expression of its downstream target gene MMP7, thereby promoting the invasion of gastric cancer cells 42. In urological malignancies, JUP primarily functions as a tumor suppressor. For instance, the developmental transcription factor SOX4 interacted with JUP, inhibiting the Wnt/β-catenin signaling pathway and reducing the expression of downstream target genes, thus hindering prostate cancer progression 43. Conversely, reduced JUP expression in prostate cancer may diminish its inhibitory effect on vascular adhesion proteins, activating the Src signaling pathway and facilitating cancer metastasis and invasion 44, 45. In the current study, we found that JUP expression was closely linked to the PI3K-AKT and MAPK pathways through RNA-seq. JUP overexpression significantly suppressed the PI3K-AKT and MAPK pathways, increased E-cadherin levels, and decreased N-cadherin and SNAIL expression. Because the interruption of contact between tumor cells may also be caused by apoptotic signals, we also examined the effects of JUP overexpression on the expression of Cleaved-Caspase 3, BAX and Bcl-2 proteins. Our findings confirmed that JUP overexpression led to a significant up-regulation of Cleaved-Caspase 3 and BAX, along with a concomitant down-regulation of BCL-2. In contrast, ASNS overexpression significantly activated the PI3K-AKT and MAPK pathways, up-regulated Bcl-2, N-cadherin, and SNAIL expression, and down-regulated Cleaved-Caspase 3, BAX, and E-cadherin. Therefore, our research results suggested that ASNS exerted its influence on PI3K-AKT, MAPK and apoptotic signals by regulating JUP. Furthermore, we demonstrated that ASNS inhibitor can significantly limited the growth and metastasis of mouse orthotopic renal tumors. However, the interaction between JUP and β-catenin and the mechanism by which it regulates the PI3K-AKT and MAPK pathways in RCC still require further exploration in the near future. Moreover, our study have indicated that ASNS overexpression suppressed VHL protein expression, whereas knockdown of ASNS promoted VHL expression; however, the underlying mechanism remains unclear. Previous studies have shown that JUP overexpression enhanced the ubiquitination of HIF-2α, leading to its instability and reduced half-life, which in turn lowered HIF-2α levels and inhibited RCC formation 46. Therefore, investigating the negative feedback regulation among the VHL/ASNS/JUP pathway is warranted, which would further strengthen the evidence supporting ASNS as a novel downstream target of VHL.

Numerous studies have demonstrated that the tumor microenvironment (TME) plays a pivotal role in tumor growth and progression, different cancer types are accompanied by distinct microenvironments, and the interactions between these environments can promote tumor progression and metastasis 47-51. Unlike other malignancies, RCC can be treated not only with surgery and targeted therapy drugs (TKIs, mTOR inhibitors, VEGF inhibitors), but also with immunotherapy, which yields remarkable therapeutic outcomes according to clinical guidelines 52-54. Furthermore, studies have indicated that TKIs can remodel the vascular networks or immune components within the TME. Therefore, combining targeted therapy agents with immune checkpoint inhibitors may represent one of the most effective therapeutic strategies for treating patients with advanced cancer 55.

Previous studies have indicated that in non-small cell lung cancer, metastatic tumor regions with high ASNS expression promoted the formation of a lymphoid microenvironment favorable for CD8+ T cell activation, memory formation, and stemness, particularly within metastatic tumor-draining lymph nodes and in close proximity to the metastatic foci, thereby reshaping the immune landscape of both primary tumors and metastatic lymph nodes 56. In bladder cancer, depletion of ASNS significantly restricted tumor growth in a CD8+ T cell-dependent manner during *in vivo* tumorigenesis and enhanced the efficacy of immunotherapy 29. In ALL, high levels of ASNS can lead to resistance to immunotherapy, overexpression of ASNS in NALM6-GL cancer cells weakened chimeric antigen receptor (CAR)-T-cell mediated cancer cell lysis 57. In this study, our findings suggest that the ASNS inhibition is a promising therapeutic target for RCC. In the near future, we will further investigate the relationship between ASNS and the immune microenvironment of RCC. Additional animal models should be employed to validate the effects of ASNS inhibitors to enhance their clinical applicability.

## Conclusions

Our research identified L-Asn as a specific metabolite associated with *VHL-*mutant RCC, playing a crucial role in the growth and metastasis of the disease. VHL was found to regulate ASNS expression through ubiquitination, leading to a decrease in L-Asn level. The inhibition of ASNS hindered RCC growth and metastasis by enhancing JUP expression, which in turn suppressed the PI3K-AKT and MAPK signaling pathways.

## Supplementary Material

Supplementary figures.

## Data Availability

All data analyzed and produced in this study are included in this manuscript.

## Abbreviations

RCC: Renal cell carcinoma
ccRCC: Clear cell RCC
VHL: Von Hippel Lindau
L-Asn: L-Asparagine
ASNS: Asparagine synthase (ASNS)
L-ASNase: L-Asparaginase
JUP: Junction plakoglobin
TCGA-KIRC: The Cancer Genome Atlas-Kidney Renal Clear Cell Carcinoma
KIRP: Kidney Renal Papillary Cell Carcinoma‌
KICH: Kidney Chromophobe Carcinoma‌
IP: Immunoprecipitation
TMT: Tandem Mass Tags
RNA-seq: RNA sequencing
OS: Overall Survival
DFS: Disease Free Survival
