# Supplementary material for: Suppression of ASNS expression by VHL-mediated ubiquitination hinders the progression of renal cell carcinoma through enhancing JUP expression and inhibiting PI3K-AKT and MAPK pathways
Source: Int J Biol Sci. 2026 Feb 26;22(6):3048–69. doi: 10.7150/ijbs.129332 (PMC13050451; doi:10.7150/ijbs.129332)

Supplemental Figure 1: ELISA expression detection of LysoA, Indole sulfate, L-Malic acid, and Ethyl glucuronide in the plasma of *VHL*-mutant RCC patients

Supplemental Figure 2：Supplemental experimental results of Fig.2F

Supplemental Figure 3: Evaluation of the clinical relevance of ASNS using TCGA-KIRC data

(A) Analysis of ASNS expression levels in ccRCCs compared to their AN tissues, along with its correlation to various tumor stages, grades, and metastasis. (B) Comparison of overall survival (OS) and disease-free survival (DFS) in ccRCC patients categorized by high and low ASNS expression. (C) Comparison of OS in metastatic ccRCC patients based on ASNS expression levels. (D) Comparison of ASNS mRNA expression between *VHL*-mutant ccRCC and *VHL* wild-type ccRCC. (E) Prognostic analysis results for patients with *VHL*-mutant ccRCC.

Supplemental Figure 4: Evaluation of the clinical relevance of ASNS using TCGA-KIRP and TCGA-KICH data

(A) Comparative analysis of the expression level of ASNS in pRCC compared to their AN tissues, as well as its relationship with various tumor stages, lymph node invasion, distant metastasis, and the OS and DFS of patients. (B) Comparative analysis of the expression level of ASNS in chRCC compared to their AN tissues, as well as its relationship with various tumor stages, lymph node invasion, distant metastasis, and patient OS and DFS.

Supplemental Figure 5：Supplemental experimental results of Fig.2M

Supplemental Figure 6：Supplemental experimental results of Fig.3A

Supplemental Figure 7: The impact of VHL overexpression on ASNS mRNA level in 786-O and RCC4 cell lines

Supplemental Figure 8：Supplemental experimental results of Fig.4B and C

(A) Supplemental experimental results of Fig.4B. (B) Supplemental experimental results of Fig.4C.

Supplemental Figure 9：Supplemental experimental results of Fig.7I

Supplemental Figure 10：Supplemental experimental results of Fig.7J

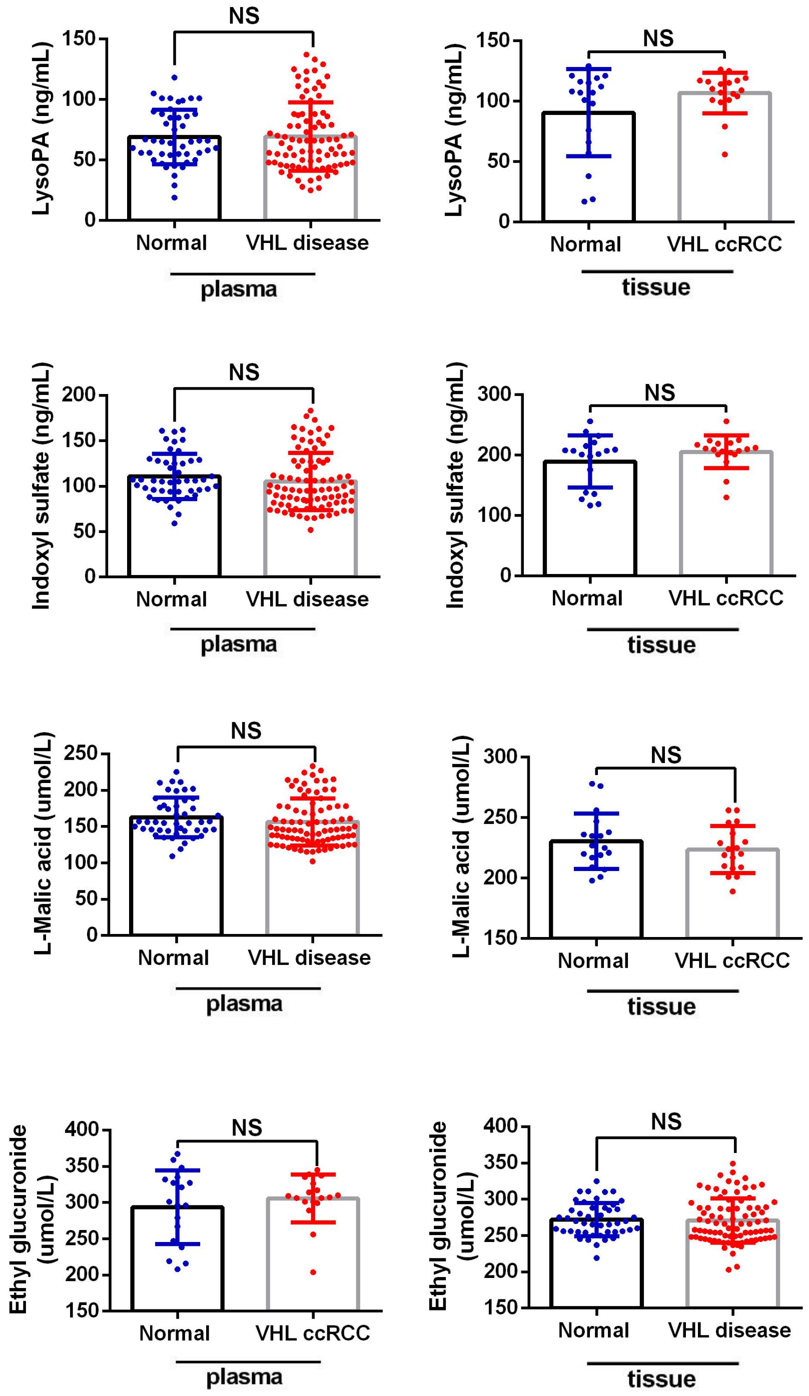
NS
LysoPA (ng/mL)
150
100
50
0
Normal
VHL disease
plasma
NS
LysoPA (ng/mL)
Normal
VHL ccRCC
tissue
NS
Indoxyl sulfate (ng/mL)
200
150
100
50
0
Normal
VHL disease
plasma
NS
Indoxyl sulfate (ng/mL)
300
200
100
0
Normal
VHL ccRCC
tissue
NS
L-Malic acid (umol/L)
250
200
150
100
50
0
Normal
VHL disease
plasma
NS
L-Malic acid (umol/L)
300
250
200
150
Normal
VHL ccRCC
tissue
NS
Ethyl glucuronide (umol/L)
400
350
300
250
200
150
Normal
VHL ccRCC
plasma
NS
Ethyl glucuronide (umol/L)
Normal
VHL disease
tissue

Fig.2F repeats

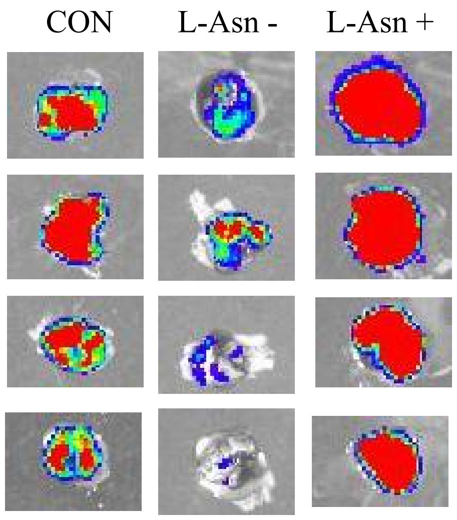

Supplemental Figure 3

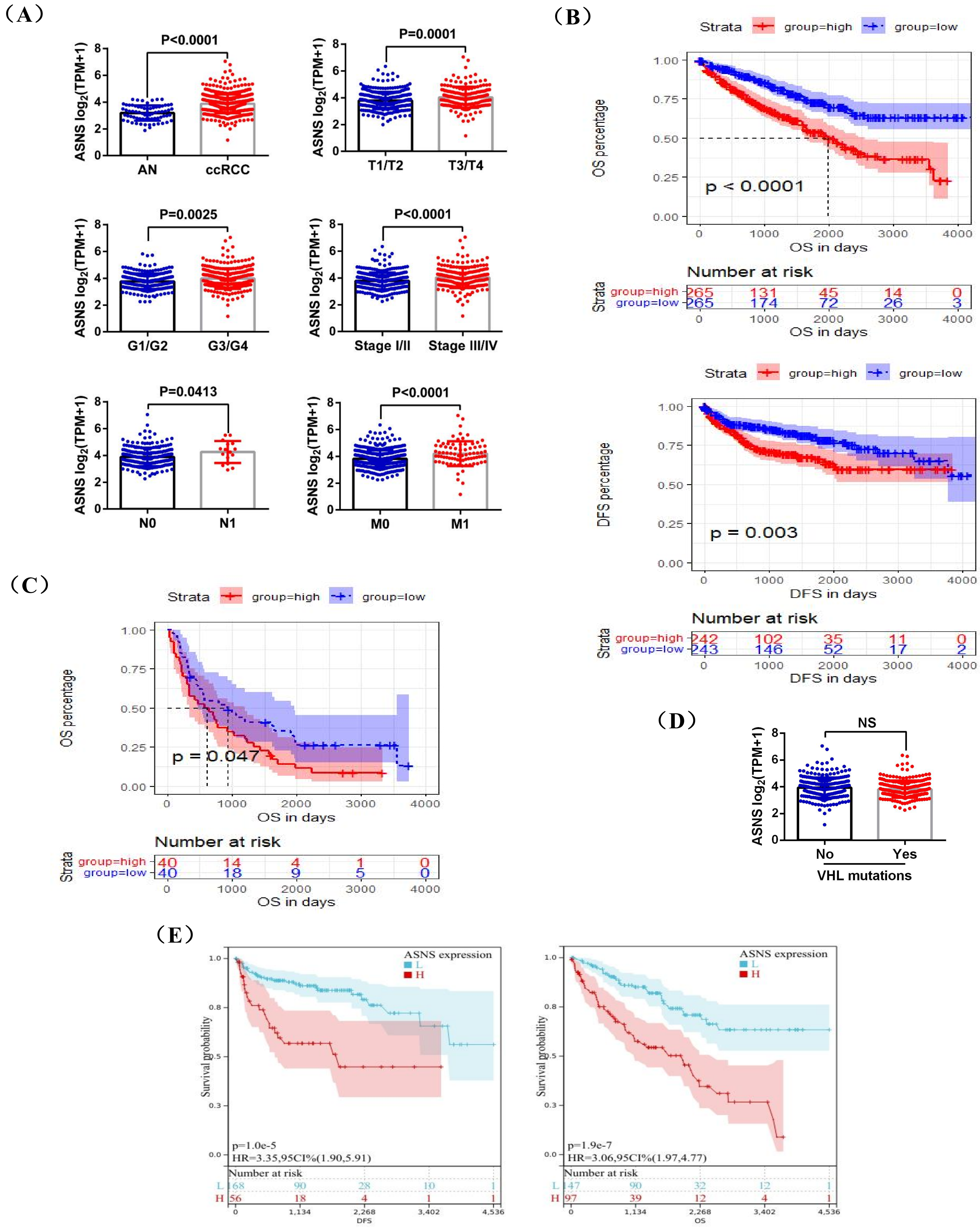

Supplemental Figure 4

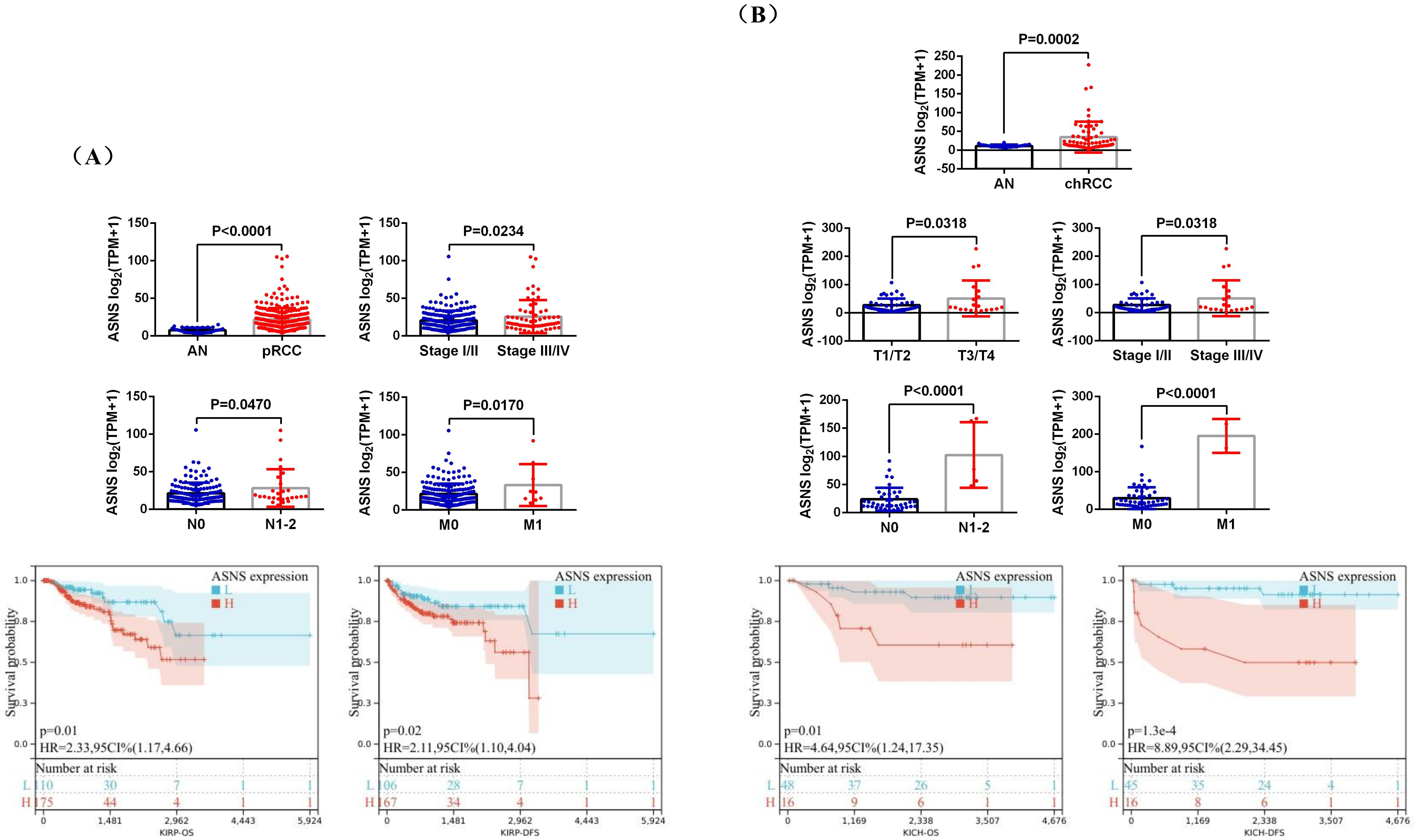

Fig.2M repeat 2, 3

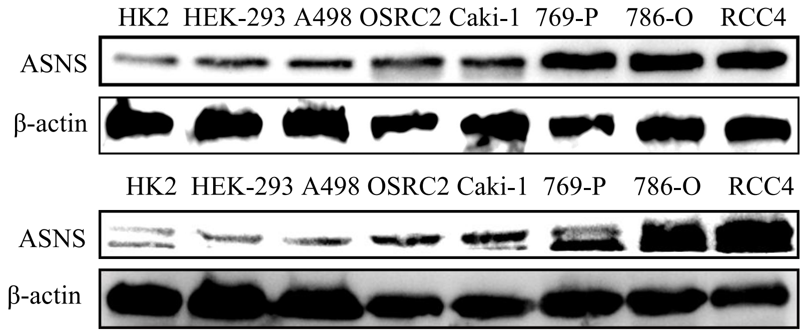

Fig.3A repeat 2, 3

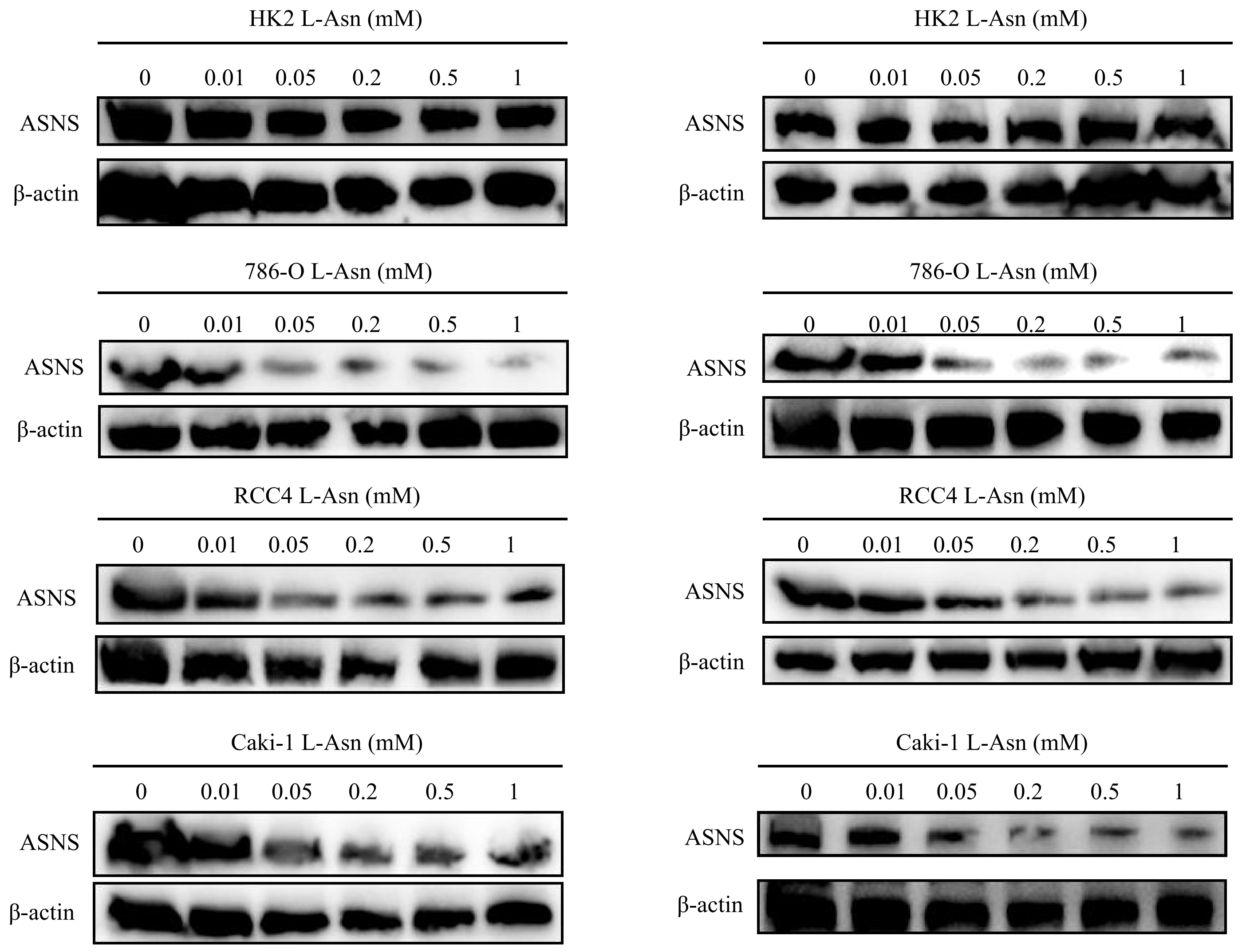

Supplemental Figure 7

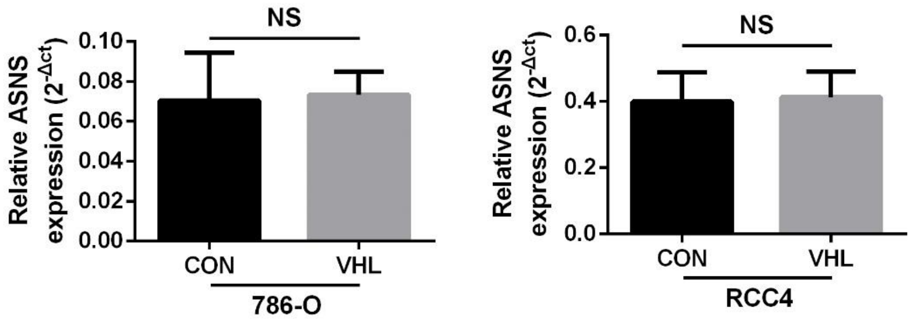

Fig.4B repeat 2, 3

(A)

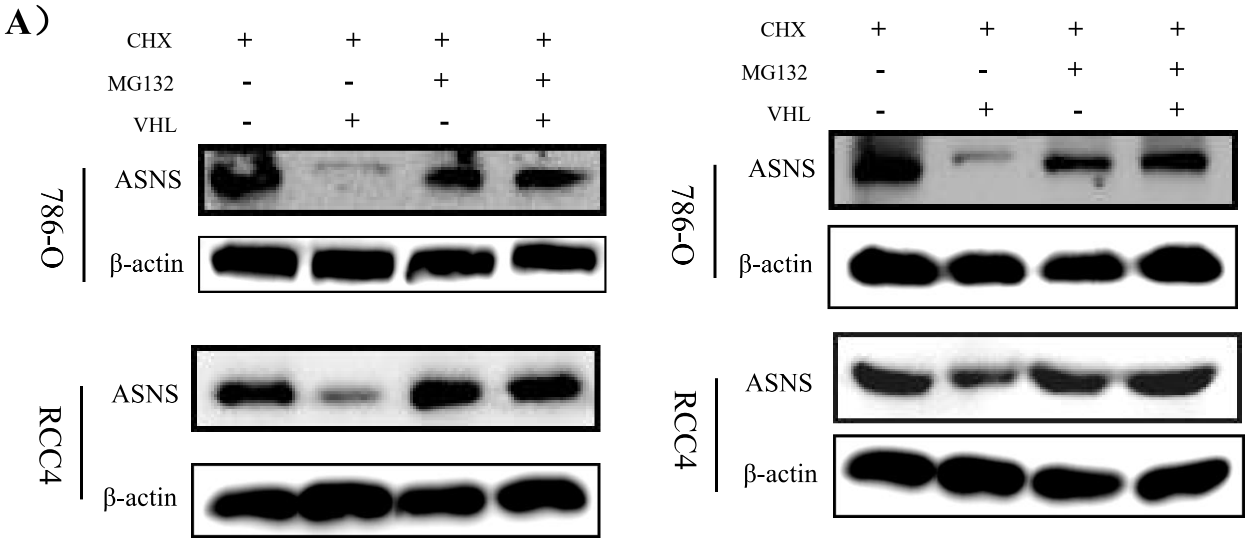

Fig.4C repeat 2, 3

(B)

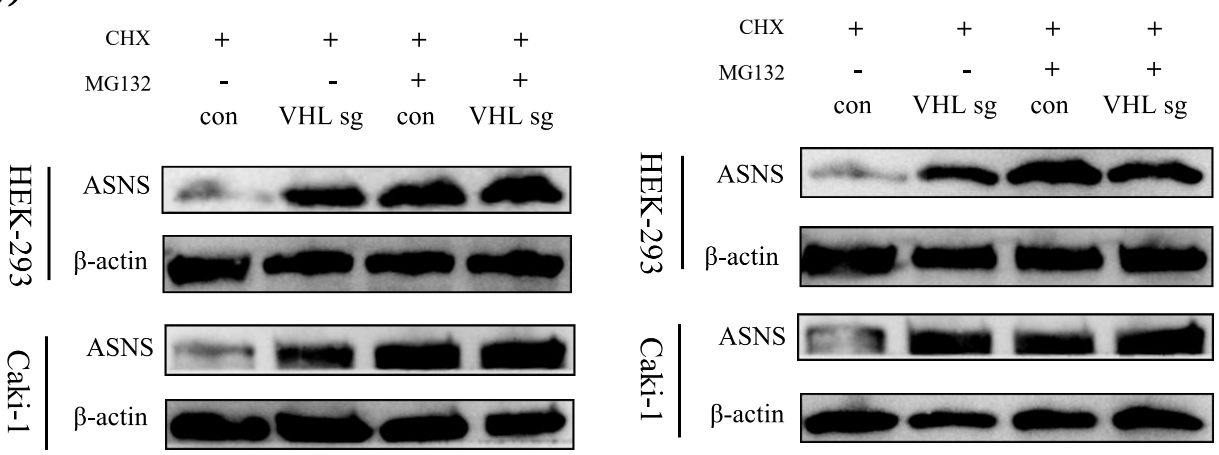

Fig.7I repeat 2, 3

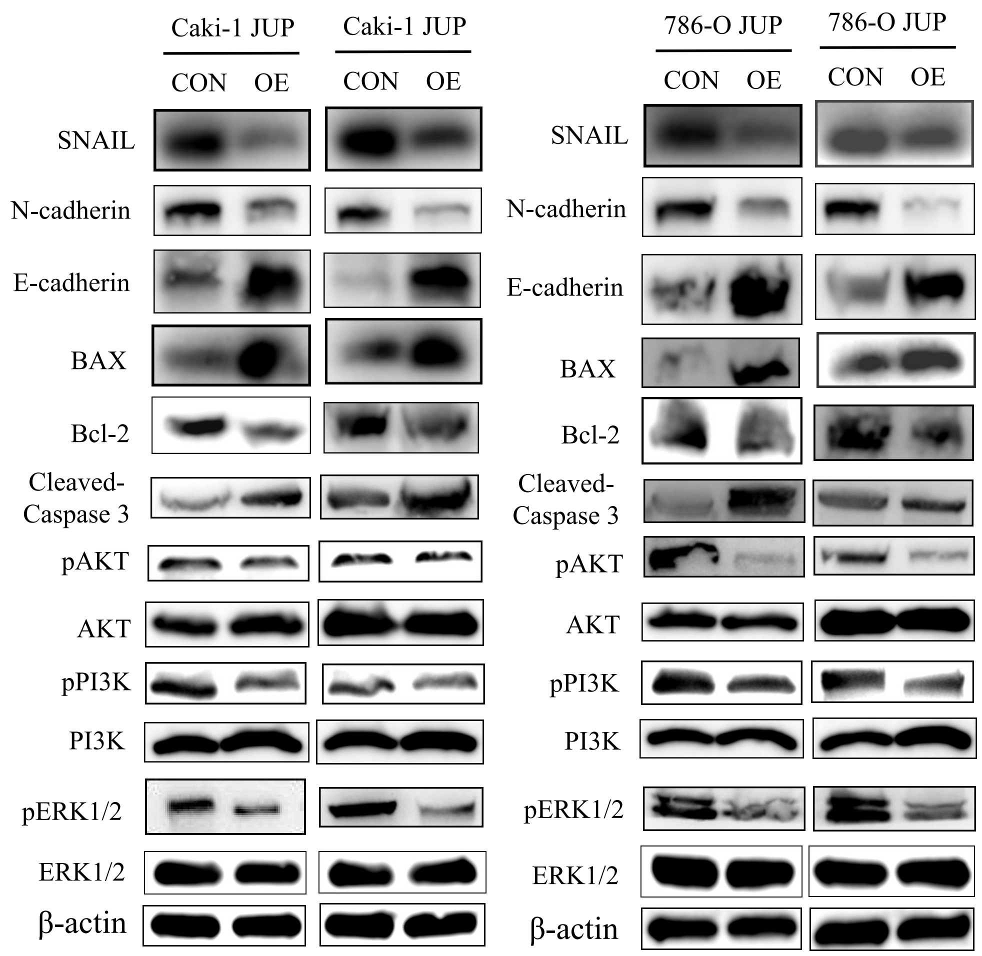

Fig.7J repeat 2, 3

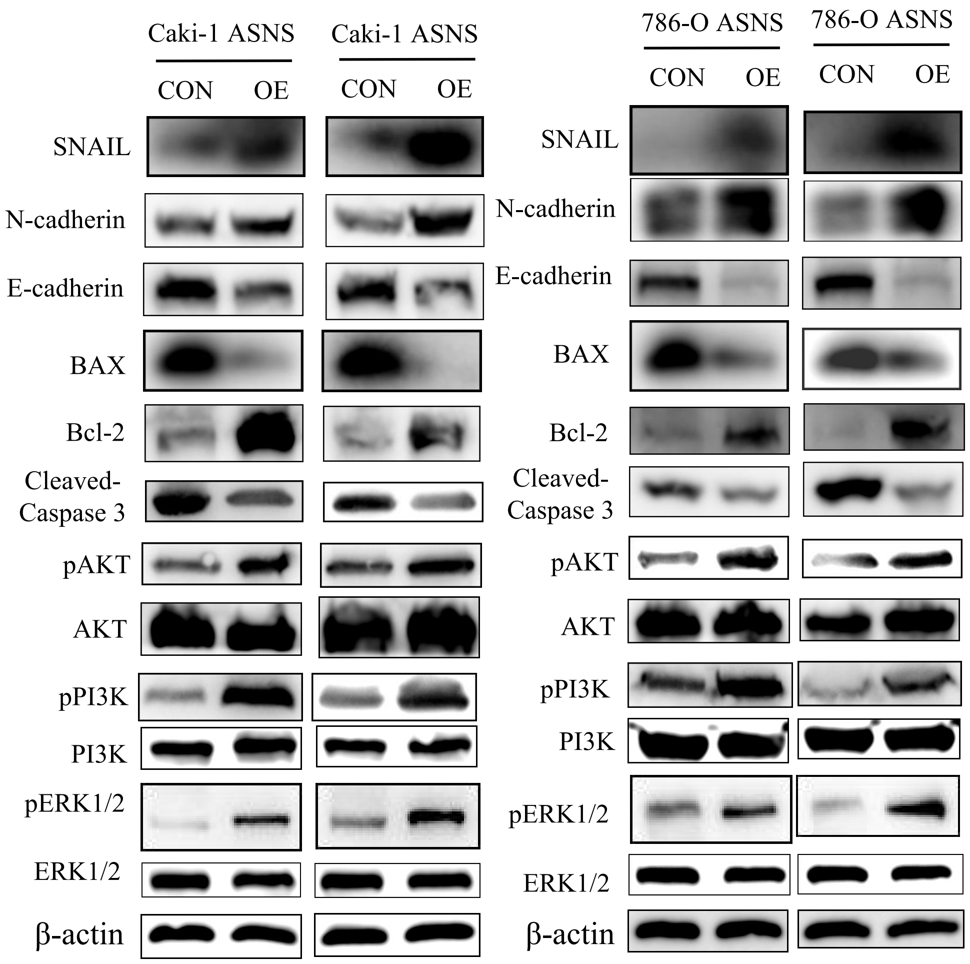

Supplement: Supplementary file 1 — Supplementary figures. [file ijbsv22p3048s1.pdf]
